# Global analyses revealed age-related alterations in innate immune responses after stimulation of pathogen recognition receptors

**DOI:** 10.1111/acel.12320

**Published:** 2015-02-27

**Authors:** Talibah U Metcalf, Rafael A Cubas, Khader Ghneim, Michael J Cartwright, Julien Van Grevenynghe, Justin M Richner, David P Olagnier, Peter A Wilkinson, Mark J Cameron, Byung S Park, John B Hiscott, Michael S Diamond, Anne M Wertheimer, Janko Nikolich-Zugich, Elias K Haddad

**Affiliations:** 1 Vaccine and Gene Therapy Institute of Florida9801 SW Discovery Way, Port St. Lucie, FL, 34987, USA; 2 Department of Pathology, Case Western Reserve UniversityCleveland, OH, 44106, USA; 3 Lady Davis Institute, Jewish General Hospital, McGill UniversityMontreal, QC, H3T 1E2, Canada; 4 Department of Medicine, Molecular Microbiology, Washington University School of MedicineSt. Louis, MO, USA; 5 Department of Pathology and Immunology, Washington University School of MedicineSt. Louis, MO, 63110, USA; 6 Division of Biostatistics, Department of Public Health and Preventive Medicine, Oregon Health and Science UniversityPortland, OR, 97239, USA; 7 Department of Immunobiology and Medicine, University of Arizona College of MedicineTucson, AZ, USA; 8 Department of the Arizona Center on Aging, University of Arizona College of MedicineTucson, AZ, 85724, USA

**Keywords:** immunosenescence, innate immune agonists, innate immunity, interferon signaling, pattern recognition receptors, peripheral blood mononuclear cells

## Abstract

Aging leads to dysregulation of multiple components of the immune system that results in increased susceptibility to infections and poor response to vaccines in the aging population. The dysfunctions of adaptive B and T cells are well documented, but the effect of aging on innate immunity remains incompletely understood. Using a heterogeneous population of peripheral blood mononuclear cells (PBMCs), we first undertook transcriptional profiling and found that PBMCs isolated from old individuals (≥ 65 years) exhibited a delayed and altered response to stimulation with TLR4, TLR7/8, and RIG-I agonists compared to cells obtained from adults (≤ 40 years). This delayed response to innate immune agonists resulted in the reduced production of pro-inflammatory and antiviral cytokines and chemokines including TNFα, IL-6, IL-1β, IFNα, IFNγ, CCL2, and CCL7. While the major monocyte and dendritic cell subsets did not change numerically with aging, activation of specific cell types was altered. PBMCs from old subjects also had a lower frequency of CD40+ monocytes, impaired up-regulation of PD-L1 on monocytes and T cells, and increased expression of PD-L2 and B7-H4 on B cells. The defective immune response to innate agonists adversely affected adaptive immunity as TLR-stimulated PBMCs (minus CD3 T cells) from old subjects elicited significantly lower levels of adult T-cell proliferation than those from adult subjects in an allogeneic mixed lymphocyte reaction (MLR). Collectively, these age-associated changes in cytokine, chemokine and interferon production, as well as co-stimulatory protein expression could contribute to the blunted memory B- and T-cell immune responses to vaccines and infections.

## Introduction

In the United States, the number of individuals over age 65 is estimated to increase to 20% of the total population by the year 2040 (Kinsella & Wan, 2009). Aging has been linked to increased susceptibility to many infectious diseases including influenza, West Nile virus (WNV), and bacterial pneumonia (Gavazzi & Krause, 2002). Unfortunately, many vaccines used in preventive strategies (e.g., seasonal influenza vaccine) do not induce an effective immune response in the elderly when compared to younger adults due to age-related dysfunctions in immunity, termed immunosenescence (Katz *et al*., 2004). Studies to understand the basis of immunosenescence and to enhance protective immunity in older adults are necessary to improve the health outcome for this expanding segment of the population.

An efficient immune response to invading pathogens or vaccination requires intricate interactions between innate and adaptive immune cells. Innate immune cells via pattern recognition receptors (PRRs) initiate and support adaptive immunity through virtue of their functions including phagocytosis, intracellular killing, release of pro-inflammatory and antiviral cytokines and chemokines, as well as antigen presentation and immune cell priming. The effect of ‘normal’ human aging on innate and adaptive immunity has been a challenge to define. Published data on the age-associated alterations in adaptive immunity have demonstrated a shift from naive to a memory T-cell phenotype, decreased T-cell responses to stimulation *in vitro*, oligoclonal expansion in the T- and B-cell repertoire, and reduced germinal center antigen responses (Nikolich-Zugich, 2008; Linterman, 2014). Aging has also been shown to influence the expression of multiple co-stimulatory and inhibitory molecules and their ligands on T and B cells and antigen-presenting cells, including CD80 expression on monocytes and ICOS and CTLA-4 expression on T cells (Leng *et al*., 2002; van Duin *et al*., 2007a; Canaday *et al*., 2013). The impact of aging on innate immunity is less well understood particularly in humans. Aging has been linked to shift in absolute numbers of innate immune cells (including monocytes, DCs, and NK cells), impaired recruitment of these cells to site of infection, reduced phagocytosis by monocytes/macrophages, as well as altered production of interferons, chemokines, and cytokines, and altered Toll-like receptor (TLR) expression (Gomez *et al*., 2005; Panda *et al*., 2009; Solana *et al*., 2012). These alterations suggest possible age-related dysfunction in PRRs responses; however, no clear consensus has been reached in defining age-related innate immune defects, in part due to the difficulty to extrapolate between mouse and human studies, the use of individual cell populations for analysis, and different age-related enrollment criteria used for subject selection.

In this report, we applied a comprehensive approach to evaluate the effects of a broad range of innate immune agonists on PBMCs isolated from healthy nonfrail adults (21–40 years) and old (65–93 years) subjects. Peripheral blood mononuclear cells (PBMCs) were used because they are composed of monocytes, dendritic cells, and lymphocytes (including T, B, and NK cells) and as a composite group represent a key defense against infection. Using PBMCs, we assessed the impact of aging on PRR signaling in heterogeneous population simulating the intracellular and intercellular interactions under physiological conditions. Besides treatment of PBMCs with specific PRR agonists, we enumerated DC and monocyte subsets, examined surface expression of co-stimulatory molecules by flow cytometry, analyzed transcriptome data, and measured cytokine and chemokine production in cultured supernatants. This approach allowed us to identify deficiencies in the PRR response in PBMCs from elderly subjects. Our findings represent a comprehensive analysis of the influence of human aging on PRR signaling/function and have implications for strategies to enhance the immune response in the context of infection or immunization.

## Results

### Individual PRR agonists elicited distinct transcriptional responses

To evaluate the transcriptional profiles elicited by individuals’ innate agonists on the integrity of human PRR signaling, PBMCs were isolated from healthy nonfrail adults and old donors (*n* = 8/group). Individuals with comorbid conditions including cancer (within the last 5 years for those ≥ 65 years), immunocompromising disorders and steroid usage (see Table1) were excluded, whereas inclusion criteria included controlled hypertension, occasional/tolerable ‘aching joints’ from arthritis and not taking daily NSAIDS or acetaminophen, and controlled diabetes (see Experimental procedures). For a description of the cohort from which these individuals were selected, see Table s7. Cells were stimulated for 6 and 24 hours (h) with LPS (TLR4), CLO97 (TLR7/8), poly I:C complexed with Lyovec (MDA-5/RIGI), or 5′-pppRNA complexed with Lyovec (RIG-I) (see Experimental procedures), and the scope of PRR responses to innate agonist stimulation was evaluated by microarray analysis using Illumina BeadChips (see Experimental procedures). Transcriptional profiles were generated by comparing LPS and CLO97-stimulated PBMCs to untreated PBMCs, whereas 5′-pppRNA/Lyovec and poly I:C/Lyovec-stimulated PBMCs were compared to Lyovec (a cationic transfection reagent that optimizes 5′-pppRNA and poly I:C effects by facilitating intracellular delivery)-only treated PBMCs. Differentially expressed genes (DEGs) were selected based on false discovery rate (FDR) 5% and fold change (FC) ≥ 1.3 or ≤ −1.3. These criteria were chosen based on our own validation scheme used for other published studies (Gaucher *et al*., 2008; Goulet *et al*., 2013).

**Table 1:** Description of subjects used for gene array, cytokine analysis, and surface phenotypic analysis

	Adult (*n *=* *8)	Old (*n *=* *8)	
Average age (range)	29 (22–38)	73 (66–85)	
Gender M/F (female %)	4/4 (50%)	4/4 (50%)	
Race			
White (non-Hispanics)	1	7	
White (Hispanics)	4		
African American	1	1	
Others (non-Hispanics)	2		
Comorbidities	3 (38%)	5 (63%)	
None	5 (63%)	3 (38%)	
Hypothyroid	1		
Diabetes mellitus	1	1	
Arthritis	1	4	
Hypertension		4	
Medications			
Over the counter	0 (0%)	1 (12.5%)	
Prescription	2 (25%)	7 (87%)	
	Furosemide	Actos	Lisinopril
	Metformin	Allopurinol	Metoprolol
	Simvastatin	Amlodipine	Nifedipine
	Synthroid	Digoxin	Nitro Cap
		Exforge	Omeprazole
		Flomax	Oxybutynin
		Lasix	Triam/HCTZ
		Lipitor	Zoloft

**Fig 1:**
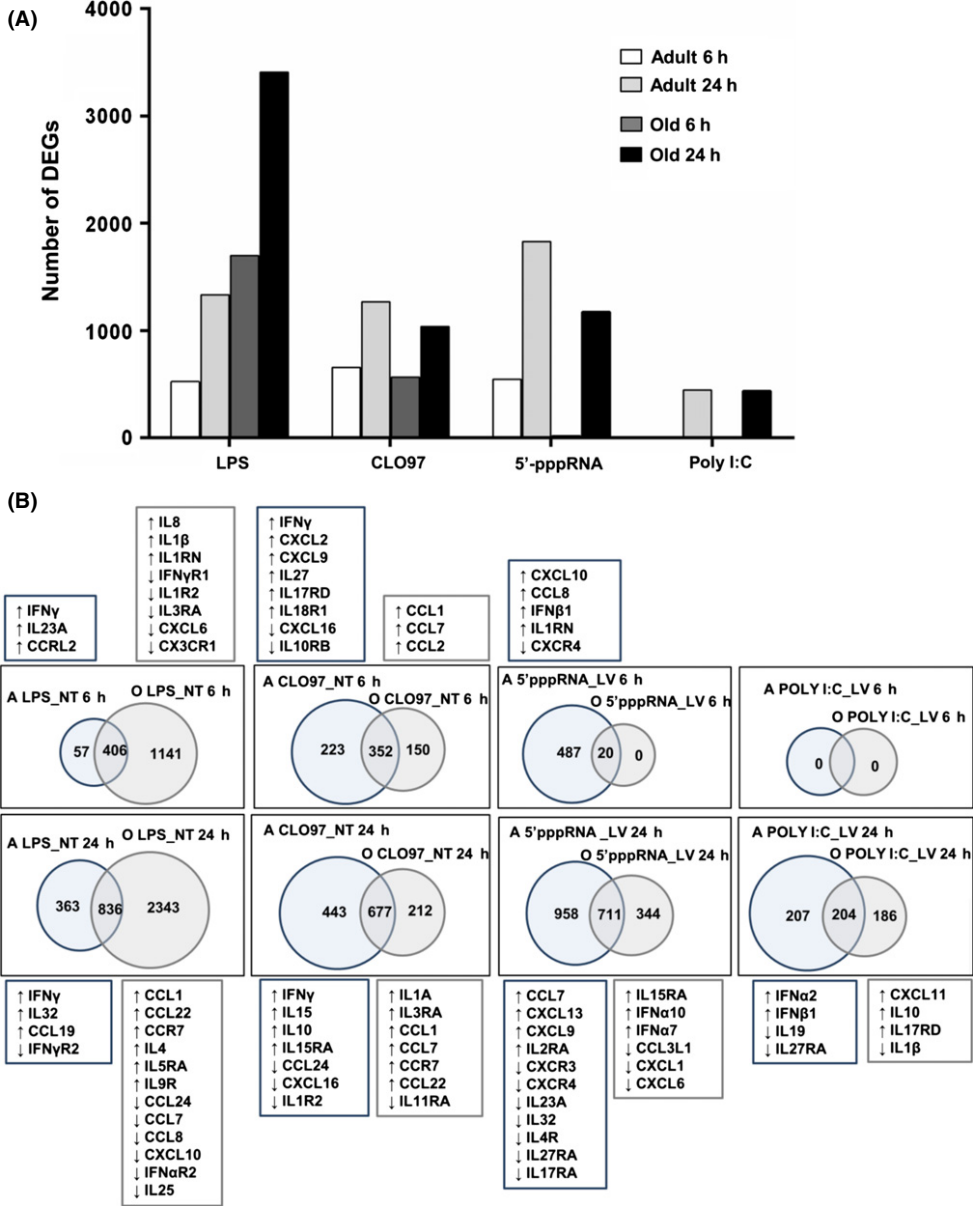
Single-gene analysis shows unique age-related differences in immune responses to PRR agonists. Agonist-treated cells (LPS, CLO97, poly I:C/Lyovec, or 5′-pppRNA/Lyovec) from adult and old subjects (*n* = 8/group) were normalized to their respective negative controls (untreated or Lyovec only treated). Gene array analysis was performed using an Illumina platform, and differentially expressed genes (DEGs) were selected to have a FDR 5% with FC ≥ 1.3 or ≤ −1.3. (A) Histograms depict the kinetics of expression and number of DEGs for agonist-treated adult and old subjects for 6 and 24 h. (B) Venn diagram analysis of agonist-treated adult (A) compared to treated old (O) subjects at 6 (top row) and 24 h (bottom row). Diagrams show the number of unique and common statistically significant genes based on the above criteria. Listed above and below each diagram are cytokines and chemokines that are uniquely elicited by each agonist at 6 and 24 h, respectively. Upregulated and downregulated genes in respect to negative controls are depicted by up- and downward arrows, respectively.

The use of sixteen subjects (*n *=* *8/group) for gene array analysis was sufficient as it yielded discriminating gene expression profiles with high number of DEGs with stringent *P* values. The number of DEGs was higher for LPS, CLO97, and 5′-pppRNA/Lyovec when compared to poly I:C/Lyovec (Fig.1A). The FDR, FC, and *P* value of all DEGs resulting from stimulation for each agonist are listed in Table s1 (adult) and S2 (old). Differentially expressed genes in response to each agonist steadily increased through 24 h in both age groups. At 24 h, higher numbers of DEGs were observed in response to 5′-pppRNA/Lyovec in adults when compared to old individuals (1828 vs. 1176), whereas the number of DEGs was higher in response to LPS in old relative to adult individuals (3410 vs. 1335). Similar number of DEGs was observed in response to CLO97 (1268 vs. 1037) and poly I:C/Lyovec (447 vs. 439) in both age groups.

### Single-gene analysis shows unique age-related differences in innate immune responses to PRR agonists

We determined whether aging impacted the immune response to agonists by comparing the transcriptional profiles of adult and old individuals. We focused our analysis on innate immunity using an immune gene filter (containing over 4000 genes) derived from Gene Ontology (GO:0045087) (Ashburner *et al*., 2000; Carbon *et al*., 2009). We first compared the untreated response of PBMCs from adult and old individuals at 6 h to determine whether there was an intrinsic difference independent of stimulation. We observed a marginal difference in untreated gene expression with only 71 genes differentially expressed at higher levels in PBMCs from adults relative to old individuals (data not shown). And only 16 of these transcripts (*HERC5, IFIT1/2, IRAK3, ISG15, MX1*, *OAS2/3, DHX58, SLAMF7, CCL2, CCL22, IFITM1/2/3,* and *IL10RB*) were annotated as immune response-related genes using the DAVID functional genomic database (da Huang *et al*., 2009). These small differences in the expression of immune genes suggest that observed differences between adult and old subjects were due primarily to their response to agonist stimulation.

Using a Venn diagram analysis, we examined the unique and common responses elicited by LPS, CLO97, 5′-pppRNA/Lyovec, and poly I:C/Lyovec for both age groups (Fig.1B). We compared transcriptional profiles (normalized to negative controls) of adult to old subjects, and using the innate immune gene filter, we identified unique immune-related DEGs for each age group (Fig.1B). Table s3 lists the unique and common DEGs resulting from Venn diagram analysis of adults to old individuals. At 6 h, relative to old individuals, PBMCs from adults appear uniquely to induce transcriptional signatures associated with pro-inflammatory chemokines and antiviral mediators that promote activation and trafficking of immune cells, including *IFN*γ and *IL23A* elicited by LPS; *IFN*γ*, CXCL2*, *CXCL9*, *IL27,* and *IL17RD* elicited by CLO97; and *CXCL10*, *CCL8,* and *IFN*β*1* elicited by 5′-pppRNA/Lyovec. At 24 h, PBMCs from adults stimulated with LPS and CLO97 continued to upregulate *IFN*γ; similarly, poly I:C/Lyovec stimulation uniquely elicited *IFN*α*2* and *IFN*β*1* only in samples from adult but not old individuals. Moreover, cells from old subjects showed upregulation of unique cytokine transcripts including *CCL1, CCL22, CCR7, IL4, IL5RA,* and *IL9R* for LPS; *IL1A, CCL1, CCL7, CCR7,* and *CCL22* for CLO97; and *IL15RA, IFN*α*10,* and *IFN*α*7* for 5′-pppRNA/Lyovec; and *CXCL11, IL10, IL17RD,* and *IL1*β for poly I:C/Lyovec (Fig.1B and Table s3). Overall, adult subjects displayed higher and sustained expression levels of multiple interferon (IFN) genes as well as unique expression of effector cytokine and chemokine genes.

### Pathway analysis reveals an age-related delay in immune responses to PRR agonists

**Fig 2:**
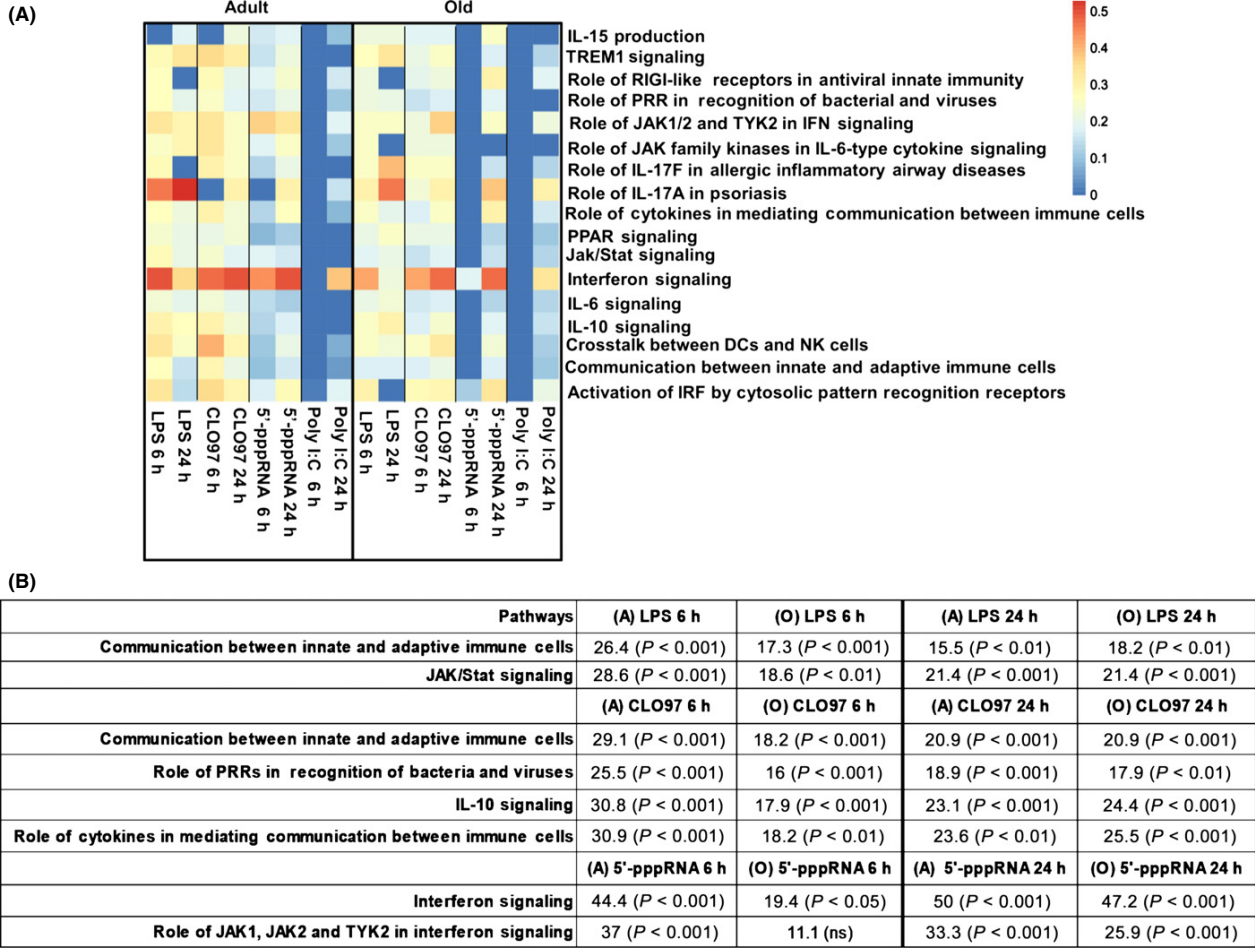
Adults show a faster and greater enrichment of functional pathways relative to old subjects. (A) Transcriptional profiles for adult and old individuals (*n *=* *8/group) generated by comparing agonist-treated PBMCs (LPS, CLO97, poly I:C/Lyovec, or 5′-pppRNA/Lyovec) to negative controls (untreated or Lyovec only treated) were analyzed by IPA canonical pathway database. Genes undergoing pathway analysis passed nominal *P *<* *0.05 and FC ≥ 1.3 or ≤ −1.3 statistical cutoffs. Heat map depicts enrichment of a custom selected list of canonical pathways (*y*-axis) at different contrasts (*x*-axis). Pathways are considered statistically significant (*P *<* *0.05) if they pass a threshold score > 0.15 (indicating 15% gene enrichment) based on a Fisher's test. Scale ranges from blue (pathway not significantly enriched, score < 0.15) to orange (significantly enriched pathway). (B) Table shows enrichment scores and *P* values of selected pathways elicited by LPS, CLO97, and 5′-pppRNA/Lyovec compared to negative controls, which shows the delay response at 6 h in old subjects.

To gain a deeper insight into age-related changes in immune gene expression, we performed a pathway-based analysis using ingenuity pathway analysis (IPA). We evaluated whether expression of disparate canonical pathways was induced in PBMCs from adult and old individuals. We determined significant enrichment of a particular pathway by calculating the numbers of genes in that pathway that were differentially expressed (*P* value < 0.05 and FC ≥ 1.3 or ≤ −1.3) in response to a particular stimulus compared to untreated or Lyovec-treated only PBMCs. Pathway enrichment scores indicate the percentage of DEGs in a specific pathway (for example, 0.15 indicates 15% of genes were differentially expressed). The pathway heat map shows the enrichment scores of immune-related pathways selected from the top forty functional pathways (Fig.2). Table s4 lists the pathway enrichment scores, *P* values, and enriched genes for each agonist.

Overall, LPS and CLO97 stimulated the largest number of enriched pathways in both adult and old subjects, whereas *interferon signaling* was the highest enriched functional pathway for all tested agonists in both age groups (Fig.2A). We observed a higher enrichment of the IFN signaling pathway following 6 h stimulation with 5′-pppRNA of PBMCs from adults (44% *P* < 0.001) when compared to those from old individuals (19.4% *P* < 0.05) (Fig.2B). This reflected a delay in IFN responses in old individuals, because there was no difference in enrichment 24 h poststimulation (adult 50% vs. old 47.2%) (Fig.2B). The induction of *the role of JAK1, JAK2, and TYK2 in interferon signaling* pathway also was delayed in PBMCs from old compared to adult individuals following stimulation with 5′-pppRNA/Lyovec at 6 h (11.1% *P* < 0.05 and 37% *P* < 0.001, respectively), but not at 24 h (25.9% and 33.3% enrichment) (Fig.2B). Other functional pathways that were more enriched in adults after 6 h stimulation included *the Jak/Stat signaling* (28.6% *P* < 0.001 vs. 18.6% *P* < 0.01) following LPS stimulation (Fig.2B). This pathway was again restored in old individuals to similar levels to adults after 24 h stimulation (both at 21% enrichment). Similarly, CLO97 stimulation elicited higher enrichment of pathways in adults [including *the communication between innate and adaptive immune cells* (29% vs. 18%), *IL-10 signaling* (31% vs. 18%), and *the role of cytokines in mediating communication between immune cells* (31% vs. 18%)], compared to old donors at 6 h (Fig.2B). After 24 h, both age groups showed similar induction of these pathways further indicating that the old donors exhibited a delayed immune response to PRR agonists. We observed no significant enrichment of pathways following stimulation with poly I:C/Lyovec at 6 h, and at 24 h, we observed similar enrichment of the *interferon signaling* pathway in both age groups (39% vs. 33%) (Fig.2A).

### Contributions of immune subsets to age-related alterations in gene expression

Monocytes and dendritic cell express distinct PRRs and produce a range of pro- and anti-inflammatory cytokines and other mediators. To evaluate the impact of aging on the numbers and proportion of monocytes and DCs in the blood, we used flow cytometry staining to identify three monocyte subsets, (i) classical (CD14^+^CD16^−^), (ii) pro-inflammatory (CD14^+^CD16^+^), and (iii) nonclassical (CD14^dim^CD16^+^), and three DC subsets, (i) myeloid (m)DC1 (CD11c^+^CD1c^+^CD141^−^CD303^−^), (ii) mDC2 (CD11c^lo^CD1c^−^CD141^+^CD303^−^), and (iii) plasmacytoid (p)DCs (CD11c^lo^CD1c^−^CD141^−^CD303^+^) (Fig. S1). Using the same individuals used for gene array analysis (*n* = 8/group), our analysis indicated that the frequencies and absolute numbers of individual monocyte and DC subsets did not vary with aging (Fig. S1). In addition, we observed similar comparison of subsets between age groups when we increased the number of subjects (data not shown). We performed univariate analysis to evaluate the effect of gender and ethnicity on absolute number of monocytes and found no significant effect of these two factors on the comparable numbers between age groups (data not shown). Therefore, the delayed and altered innate immune response observed in old individuals could not be ascribed to a mere change in subset abundance. Rather, they were likely due to a decreased induction of gene expression in particular cell subsets.

**Fig 3:**
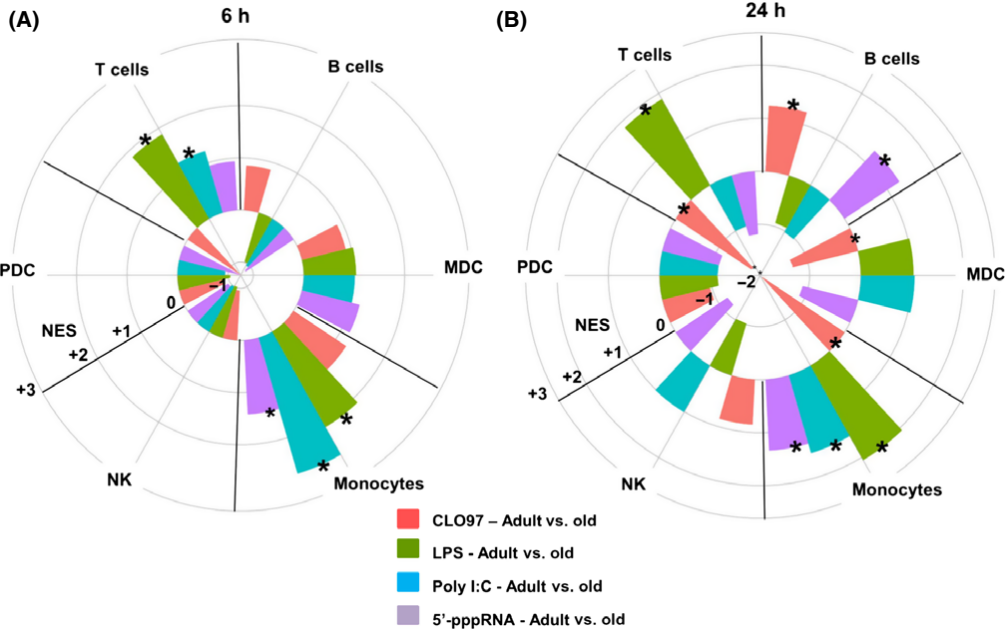
Agonist stimulation induced a monocyte cell signature in adults compared to old counterparts. Transcriptional profiles generated from comparing adult stimulated PBMCs to old stimulated PBMCs (*n *=* *8/group) were analyzed using cell type-specific modules. The radial plots illustrate the enrichment of genes associated with T and B cells, natural killer (NK), plasmacytoid (p), and myeloid (m) DCs, and monocyte subsets for a given treatment (LPS, CLO97, poly I:C/Lyovec, or 5′-pppRNA/Lyovec) at 6 (A) and 24 h (B), by plotting the Normalized Enrichment Score (NES) calculated by GSEA that takes into account the FDR using the Benjamini and Hochberg method (Subramanian *et al*., 2005). Gene sets induced in a specific cell type that was significantly enriched in adults or old subjects are denoted by asterisks: * for *P* value < 0.05. A positive enrichment score (+NES; radially outward from 0) represents upregulation in adults, whereas a negative score (−NES; inward from 0 to center) represents upregulation in old subjects.

To assess the contributions of specific cell types in the overall peripheral blood immune response signature in adult vs. old individuals, we applied an approach for deconvoluting gene expression profiles based on the meta-analysis of cell type-specific gene expression signatures from publicly available microarray studies (Nakaya *et al*., 2011). A positive normalized enrichment score (+NES) represents upregulation in adults, whereas a negative score (−NES) represents upregulation in old subjects (Fig.3). Table s5 lists the specific cell type NES, *P* values, and enriched genes for each agonist.

After 6 h of LPS, 5′-pppRNA/Lyovec, or poly I:C/Lyovec stimulation, PBMCs from adults induced genes that were preferentially expressed by monocytes (+NES 2.11, 1.42, and 2.72, respectively), whereas LPS and poly I:C/Lyovec induced genes specific to T cells (+NES 1.87 and 1.23, respectively) when compared to cells from older individuals (Fig.3A). By 24 h of stimulation, PBMCs from adults induced immune genes that were specific to three cell types: (i) monocytes in response to LPS (+NES 2.06), 5′-pppRNA/Lyovec (+NES 1.34), and poly I:C/Lyovec (+NES 1.53), (ii) T cells in response to LPS (+NES 1.87), and (iii) B cells in response to CLO97 (+NES 1.24) and 5′-pppRNA (+NES 1.28). For PBMCs from old subjects, gene induction of cellular subsets following stimulation was observed only after CLO97 treatment at 24 h, which upregulated genes associated with T cells (−NES −1.76), mDCs (−NES −1.35), and monocytes (−NES −1.92) (Fig.3B). Overall, genes corresponding to monocytes were strongly induced at an early time after PRR agonist treatment in PBMCs from adult subjects. Moreover, cells from adult subjects exhibited greater responsiveness within specific cellular subsets suggesting that aging limits PRR responsiveness in several different cell types. Importantly, similar frequency of monocytes (Fig. S1) with comparable expression of TLR3 (mainly recognize naked poly I:C and to some extent poly I:C complexed with Lyovec), TLR4 (LPS), and TLR7 (CLO97) (Fig. S2) was observed between age groups; thus, the delayed monocyte response in old individuals was not due to lower TLR expression.

### Age-associated alteration in surface expression of immune regulatory molecules

**Fig 4:**
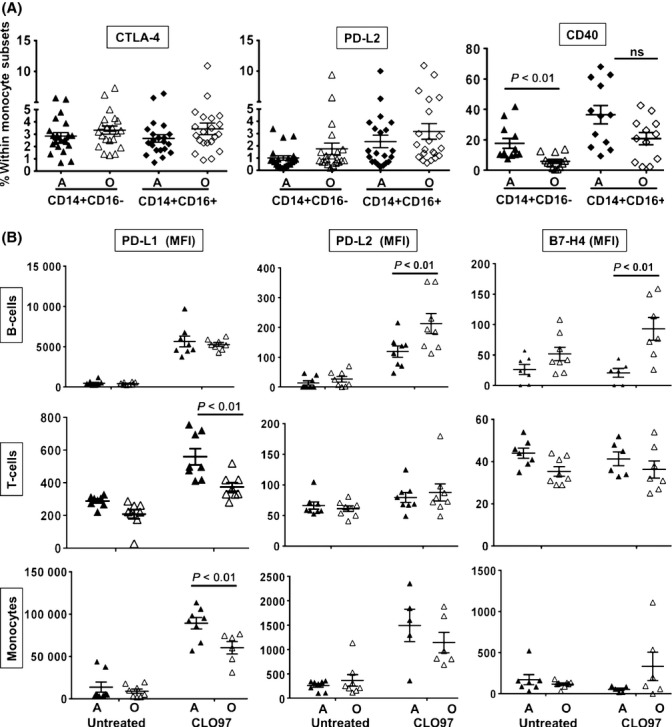
Age-related changes in surface expression of co-stimulatory molecules. (A) PBMCs were stained for CD3+ T cells, CD19+ B cells, CD14+ monocytes, and either CTLA, PD-L2, or CD40. CTLA and PD-L2 (*n *=* *21/group) and CD40 (*n *=* *12/group). Individual values along with mean ± SEM are shown. *P* values were determined by comparing adult to old subjects using the Mann–Whitney *U*-test. (B) PBMCs isolated from adult and old subjects (*n *=* *8/group) were stimulated with CLO97 for 24 h. PBMCs were stained for CD3+ (T cells), CD19+ (B cells), CD14+ (monocytes), and either PD-L1, PD-L2, or B7-H4. MFI values along with mean ± SEM are shown. Statistical significance between adult (A) and old (O) subjects was determined by two-way ANOVA followed by Sidak correction for multiple comparisons. For untreated vs. treated, *P* values are not shown for significant upregulation: PD-L1 on monocytes and B cells from both age groups (*P* < 0.001) and on T cells from A (*P* < 0.001) and O (*P* < 0.01); PD-L2 on monocytes from A (*P* < 0.001) and O (*P* < 0.01) and B cells from A (*P* < 0.01) and O (*P* < 0.001); B7-H4 on B cells from O (*P* < 0.05).

To further define the significance of age-related changes after PRR stimulation, we measured the basal expression levels of key molecules (e.g., CTLA-4, PD-L2, and CD40) that regulate immune cell activation (Fig.4A). The frequencies of CD14+CD16-CD40+ monocytes (17.7% vs. 5.9% *P *<* *0.01) and CD40+ B cells (7.5% vs. 4.4% *P *<* *0.05) were higher in adults compared to old subjects (Fig.4A and S3). However, we did not detect a difference in the surface expression of inhibitory molecules on monocytes (CTLA-4 and PD-L2) or DCs (PD-L1, PD-L2, and CTLA-4) with aging (Fig.4A and data not shown).

The expression of regulatory molecules (including ICOS, CTLA-4, PD-L1, PD-L2, and B7-H4) on peripheral blood subsets also was examined in response to TLR7/8 stimulation. For both age groups, PD-L1 was significantly upregulated on monocytes, T cells, and B cells and PD-L2 was upregulated on monocytes and B cells, whereas B7-H4 was upregulated only on B cells from old subjects (Fig.4B). At 24 h, PD-L1 expression was greater on the surface of CD14+ monocytes from adults (mean fluorescence intensity (MFI) 92 136 vs. 60 073 *P *<* *0.01) and CD3+ T cells (MFI 560 vs. 374 *P* < 0.01) compared to old individuals (Fig.4B). However, B cells from old subjects had higher expression of inhibitory molecules PD-L2 (MFI 213 vs. 113 *P *<* *0.01) and B7-H4 (MFI 93 vs. 21 *P *<* *0.01) (Fig.4B). No difference in expression levels of ICOS and CTLA-4 was detected on any cell type between adult and old subjects (data not shown). These results suggest that aging alters the surface expression of key regulatory molecules on innate and adaptive immune cells, which could contribute to the dysfunctional immune response observed in the elderly.

### PBMCs from adults produced higher levels of inflammatory cytokines, IFNs, and chemokines in response to PRR agonists

To assess whether the observed delayed transcriptional immune response in PBMCs from old individuals translated into lower production of cytokines and chemokines, we profiled supernatants from PRR-stimulated PBMCs used in gene array analysis. Supernatants were initially analyzed by ELISA and cytometric bead array kits (CBA); however, to gain a more global analysis of multiple cytokine/chemokine production, supernatants were analyzed using Milliplex bead assays. Table s6 lists concentrations of measured cytokines and chemokines elicited by each agonist. At 24 h, we observed the highest induction of cytokines and chemokines following the stimulation of TLR7/8 including IFNα, IFNγ, TNFα, IL-1β, IL-6, IL-1RA, IL-10, CCL1, CCL2, CCL3, CCL4, CCL7, CXCL1, CXCL10, CX3CL1, and VEGF. In comparison, TLR4 stimulation induced secretion of IFNα, TNFα, IL-1α/1β, IL-2, IL-6, IL-13, IL-23, IL-1RA, IL-10, CCL1, CCL3, CCL4, CCL22, CXCL1, CX3CL1, VEGF, G-CSF, GM-CSF, and Flt-3L, whereas RIG-I activation induced the expression of IFNα, CCL7, CCL8, CXCL5, and CXCL10. Poly I:C stimulation failed to elicit a measurable cytokine response.

**Fig 5:**
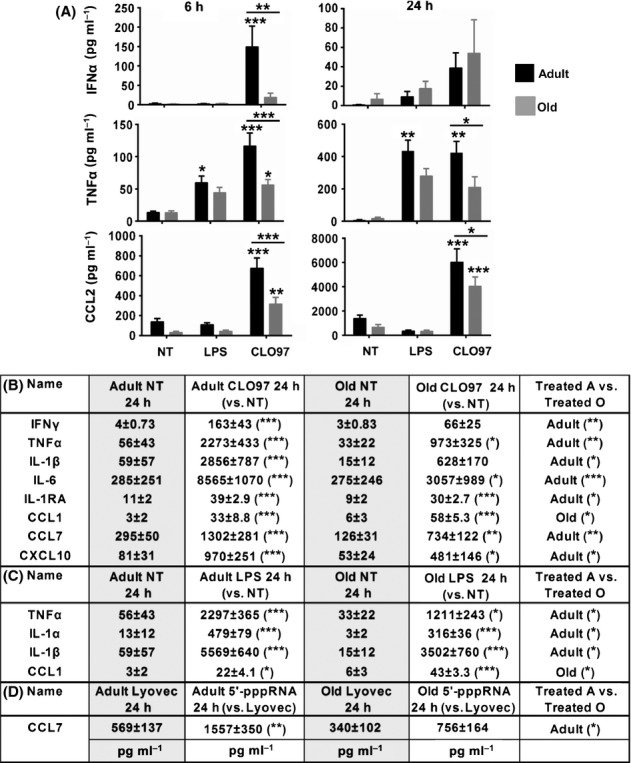
PBMCs from adults produced higher levels of inflammatory cytokines, IFNs, and chemokines in response to PRR agonists. (A) Graphs depict the means ± SEM of IFNα, TNFα, and CCL2 concentrations in response to stimulation with CLO97 and LPS for 6 and 24 h for adult and old subjects. TNFα and CCL2 were measured by CBA, whereas IFNα was measured by ELISA. (B-D) Table depicts the means ± SEM for cytokines and chemokines measured by Milliplex that showed age-related alternations after stimulation with (B) CLO97, (C) LPS, and (D) 5′-pppRNA/Lyovec for 24 h. Asterisks indicate statistical significance (***) *P* < 0.001, (**) *P* < 0.01, and (*) *P* < 0.05) between treated PBMCs vs. negative controls and adult vs. old subjects using two-way ANOVA followed by Sidak correction for multiple comparisons. *n *=* *8/group.

A comparison of adults to old subjects revealed age-related alternation in the production of IFNα (149 vs. 19 pg mL^−1^ *P* < 0.01), TNFα (116 vs. 56 pg mL^−1^ *P* < 0.001), and CCL2 (677 vs. 317 pg mL^−1^ *P* < 0.001), which was higher in PBMCs from adults treated with CLO97 for 6 h (Fig.5A). IFNα was restored in old subjects by 24 h after stimulation with CLO97, whereas cells from adults still produced higher levels of TNFα and CCL2 (Fig.5A). Our results at 24 h showed PBMCs from adults in response to CLO97 also produced higher levels of TNFα, IFNγ, IL-1β, IL-6, IL-1RA, CCL7, and CXCL10 (Fig.5B). Peripheral blood mononuclear cells from adults stimulated with LPS produced higher levels of TNFα, IL-1α, and IL-1β (Fig.5C) and, when stimulated with 5′-pppRNA/Lyovec, produced higher levels of CCL7 (Fig.5D). CCL1 was the only measured inflammatory mediator that was higher in the supernatants in response to LPS and CL097 in old subjects at 24 h (Fig.5-C). This result corroborated the unique induction pattern of the CCL1 transcript in old subjects following stimulation with LPS and CLO97 (Fig.1B). Notably, we found similarity between mRNA and protein levels for some genes including IFNγ and CCL7, whereas others genes such as CCL2, TNFα, and IL-1β which was altered by aging at the protein levels were found to be unchanged at the transcript levels. This could arise from the kinetics of expression or biological factors, such as mRNA degradation. Overall, the delayed transcriptional response by PBMCs from old individuals was accompanied by a decreased production of pro-inflammatory cytokines and chemokines.

### PBMCs from adults stimulated higher levels of T-cell proliferation in response to PRR agonists

**Fig 6:**
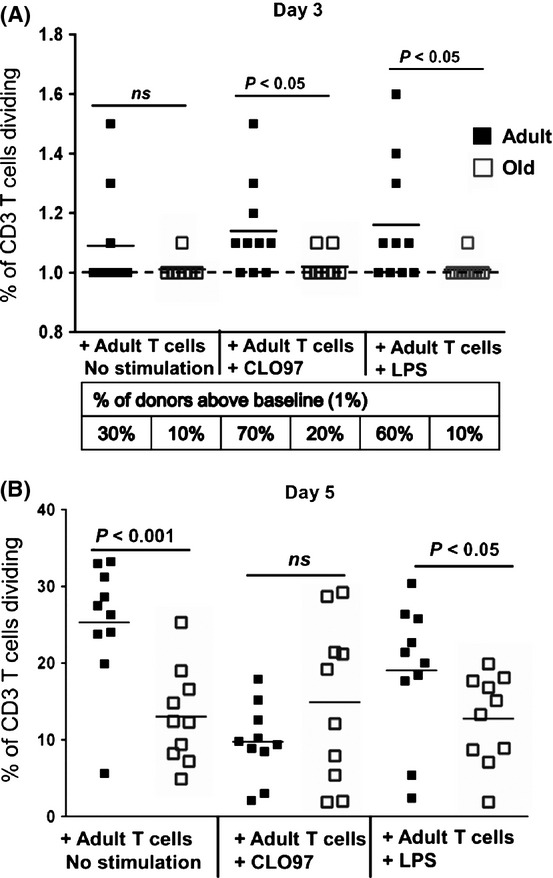
PBMCs from adults induced higher levels of proliferation of allogeneic T cells. Allogeneic mixed lymphocyte reaction (MLR) using LPS- or CLO97-stimulated PBMCs (minus CD3 T cells) from adults and old donors (*n *=* *10/group) cultured with pooled CFSE-labeled CD3+ T cells from adults (*n *=* *2). Graphs depict the frequency of dividing CD3 T cells (CFSE low) after day 3 (D3) (A) and day 5 (D5) (B) of culturing. Individual values along with mean are shown. Frequencies > 1 (baseline) were considered significant, indicating a gating of > 20 events. For D3, the percentage of donors above baseline (dash line) is listed in table below graph. Statistical significance between adults and old subjects was determined by Mann–Whitney *U*-test.

To assess whether the cross talk between innate and adaptive immunity was impaired in old adults, we performed an allogeneic mixed lymphocyte reaction (MLR) using agonist-stimulated PBMCs (minus CD3 T cells) from adults and old donors (*n *=* *10/group) cultured with pooled CFSE-labeled CD3+ T cells from adults (*n *=* *2) (Fig.6). At day 3 (D3) of coculture, in the absence of TLR stimulation, the percentage of adult donors that elicited T-cell proliferation was higher (although not significant) compared to old donors (30% vs. 10%) (Fig.6A). Pre-incubation of adult PBMCs (minus CD3 T cells) with PRR agonists further enhanced the proliferation of T cells with CLO97 inducing 70% of adults vs. 20% of old subjects (*P *<* *0.05), whereas LPS induced 60% of adults vs. 10% of old subjects (*P *<* *0.05). At D5, untreated and LPS-stimulated PBMCs from adults continued to induce higher T-cell proliferation (*P *<* *0.01 and *P *<* *0.05, respectively) (Fig.6B). These results demonstrate that aging alters the ability of innate immune cells to activate T cells, which could contribute to the dysfunctional adaptive immune responses observed in the elderly.

## Discussion

We used a global approach to study the early and late effects of a broad range of innate immune agonists on PBMCs isolated from adult and old individuals. A major finding of this study is that PBMCs from old subjects exhibited a slower immune response to PRR agonists compared to cells from adult individuals. This was evident by the rapid induction of the *interferon signaling* pathway in PBMCs from adults treated with LPS, CLO97, and 5′-pppRNA/Lyovec, as well as secretion of IFNα and IFNγ in response to CLO97. Our results support other studies that showed interferon production is impaired in PBMCs from elderly subjects stimulated *in vitro* with endotoxin or virus antigens (Abb *et al*., 1984; Ouyang *et al*., 2000). Type I IFN (IFNα/β) and type II IFN (IFNγ) induce antiviral activity and cell-mediated immunity, respectively, and regulate the differentiation of effector T-cell subsets and activation of B cells leading to a robust Ag-specific immune response (Jego *et al*., 2003; Commins *et al*., 2010). Thus, age-dependent delays in IFN signaling could contribute to heightened susceptibility to viral infections within the elderly population, such as neuro-invasive WNV (Samuel & Diamond, 2005).

This delayed interferon response also was associated with reduced production of pro- and anti-inflammatory cytokines and chemokines including TNFα, CCL2 IL-1β, IL-6, IL-1RA, CCL7, and CXCL10; however, old subjects did produce higher levels of CCL1 in response to LPS and CLO97. *In vitro* studies aimed at examining cytokine production in aging population have yielded inconsistent results. For example, it has been reported that DCs and monocytes obtained from old subjects produced more cytokines than those form adults (Alvarez-Rodriguez *et al*., 2012; Hearps *et al*., 2012). On the other hand, others have reported no change or lower production in old compared to adult subjects (Gon *et al*., 1996; van Duin *et al*., 2007b; Panda *et al*., 2010). In the case of PBMCs, studies have yielded increased production of cytokines and chemokines [including CCL3, CCL5, IL-6, TNFα, and IL-1β (Fagiolo *et al*., 1993; Pulsatelli *et al*., 2000)] in the elderly population, which is contrasting to our results. A contributing factor for these conflicting studies could be the use of different stimuli or method of preparation of the same stimulus, which can influence the immune response and level of cytokine produced. A low-grade chronic pro-inflammatory status is associated with aging (termed ‘inflamm-aging’), which appears to be more associated with comorbidities (such as frailty) and not healthy normal aging (Franceschi *et al*., 2007). Thus, different enrollment criteria (including definition of frailty) used by various studies could also explain these conflicting results. Using nonfrail subjects, our work provides further evidence for cytokine dysregulation during normal aging.

We observed the enrichment of functional pathways (including *the communication between innate and adaptive immune cells*, *roles of cytokines in mediating communication between immune cells*, *cross talk between DCs and NK cells,* and *interferon signaling*) in young subjects that suggest both innate and adaptive immunity are altered by aging in old subjects. In our study, it is difficult to show the contribution of individual immune subsets to the overall impairment observed in old subjects; however, at the transcriptional level, monocyte signal was more enriched in adults for up to 24 h in response to LPS, poly I:C/Lyovec, and 5′-pppRNA/Lyovec, as well as T-cell signal induced by LPS up to 24 h and poly I:C at 6 h and B cells induced by CLO97 and 5′-pppRNA at 24 h. The delayed and blunted levels of interferons, cytokines, and chemokines produced in old individuals could have been caused by the direct or indirect activation of both innate and adaptive immune cells. In fact, age-related decrease in IFNγ and TNFα-positive CD4+ T cells has been observed in old subjects after activation (Alberti *et al*., 2006). Therefore, it is possible that the immune response defects observed in old subjects are global and involved all arms of the immune response.

Monocytes and dendritic cells are first line of defense against invading pathogens and play a pivotal role in immune regulation by producing cytokines including IL-1α/β, IL-6, IL-8, and TNFα and presenting antigens to T cells. At the transcriptional level, we observed the signal for monocytes and pDCs enriched in adults and old subjects, respectively, but it is also difficult in our study to know the contributions of monocyte and DC subsets in the overall age-associated changes. Although aging can cause a shift in human monocyte and dendritic cell subsets (Jing *et al*., 2009; Seidler *et al*., 2010), as well as reduced TLR expression (Kong *et al*., 2008; Shaw *et al*., 2011; Qian *et al*., 2012), we observed unaltered proportions of monocytes and dendritic cells and no differences in TLR surface and intracellular expression on monocytes. Our work suggests the impairment of downstream signaling events in monocytes and dendritic cells from old donors.

Lymphocyte interaction with co-stimulatory (e.g., CD80, CD86, and CD40) and negative regulatory molecules (e.g., PD-L1, PD-L2, and B7-H4) expressed on activated innate immune cells, as well as stimulation by cytokines and chemokines, is necessary for the differentiation into effector subsets. Co-stimulatory and negative regulatory molecules are regulated by inflammatory cytokines, including type I IFN and type II IFN (Freeman *et al*., 2000; Zhu *et al*., 2012). The impaired cytokine signaling observed in old PBMCs could differentially affect expression of these regulatory molecules between adult and old individuals. Indeed, we observed age-related alternations in the upregulation of PD-L1 on T cells and monocytes and PD-L2 and B7-H4 on B cells in response to CLO97 after 24 h. We observed old individuals had reduced frequency of CD40+ monocytes and B cells compared to adults under nonactivated conditions. Previously, the percentage of CD40+ B cells was shown to be unaltered with aging (Colonna-Romano *et al*., 2003). These contrasting results could arise from the use of different culturing conditions (fresh blood verse frozen PBMCs) and different cohorts. More insight will be gained by analyzing CD40 expression on agonist-stimulated cells mimicking a pathogen response. Indeed, analyses of TLR-induced upregulation of co-stimulatory proteins on monocyte revealed age-associated defects in co-stimulatory CD80 expression but not CD86 (van Duin *et al*., 2007a).

The observed age-related alternations in cytokine and chemokine production, as well as expression of co-stimulatory and inhibitory molecules, lead us to hypothesize that the interface between the innate and adaptive immune responses is impaired in the elderly, which might explain the increased susceptibility to infectious diseases and poor responses to vaccination. We observed that agonist-stimulated PBMCs (minus CD3 T cells) from old subjects induced lower proliferation of allogeneic adult T cells compared to stimulated PBMCs from adult subjects, suggesting the ability of innate immune cells (including monocytes and DCs) to activate T cells is altered with aging. These results were supported by other studies which showed proliferative responses of T cells from elderly subjects were significantly reduced when mixed with non-T cells from adults (Molteni *et al*., 1994; You *et al*., 2013).

In summary, our results provide a comprehensive analysis of the signaling of multiple PRRs in PBMCs in the context of aging. Peripheral blood mononuclear cells from old subjects were less responsive to innate immune agonists and generated responses of altered quality and quantity. These age-related changes in the cytokine milieu and co-stimulatory molecule expression have the potential to impair the cross talk between the innate and adaptive immunity, which might explain the increased susceptibility to infectious diseases and poor responses to vaccination. Our agonist-induced functional profiles provide a basis for identifying and studying distinct genes and mechanisms important in PRR signaling. This should foster the design of new approaches to improve vaccine efficacy and antipathogen responses in the elderly population.

## Experimental procedures

### Ethic statement

This study was approved by the Institutional Review Board of the University of Arizona (Tucson, AZ) and Martin Health System (Stuart, FL, USA). All participants in this study were > 21 years of age. Written informed consent was obtained from all subjects.

### Patient recruitment and PBMC isolation

Healthy community-dwelling subjects were enrolled from University of Arizona and Martin Health system (Florida) in two groups: adults (21–40 years, *n *=* *31) and nonfrail old individuals (65–93 years, *n *=* *31). Using a screening questionnaire, participants were asked about lifestyle, clinical history, and medication usage. Subjects were excluded that self-reported comorbid conditions including cancer (within the last 5 years for those ≥ 65 years), immunocompromising disorders, and steroid usage, whereas inclusion criteria included controlled hypertension, occasional/tolerable ‘aching joints’ from arthritis, and not taking daily NSAIDS or acetaminophen, and controlled diabetes. Our definition of nonfrail subjects was based on Fried criteria (Bouillon *et al*., 2013). The Katz index of independence in activities of daily living (ADL) was used to assess the functional impairment of subjects ≥ 65 years (Katz *et al*., 1970). The index ranks adequacy of performance in bathing, dressing, toileting, transferring, continence, and feeding. Subjects with a score lower than 6 (indicating moderate to severe impairment) were excluded from the study. Old subjects were also assessed for dementia using the Mini-COG assessment instrument for dementia (Borson *et al*., 2003). The Mini-Cog combines two simple cognitive tasks [three-item word memory and clock drawing test (CDT)] with an empirical algorithm for scoring. Subjects with a recall score of 1–2 words with a normal CDT or a recall score of three words were considered nondemented. Demographic data for the cohort used in this study are described in Table s7. The description of subjects used for gene array, cytokine analysis, and surface phenotypic analysis is provided in Table1 (*n *=* *8/group). For some surface phenotypic analysis and MLR analysis, additional subjects were selected from the cohort. Blood was drawn into heparinized Vacutainer CPT tubes (BD Bioscience, Franklin Lakes, NJ, USA) and processed as per manufacturer's recommendations to isolate peripheral blood mononuclear cells (PBMC) and plasma; Li-EDTA tubes were used to determine complete blood counts. Peripheral blood mononuclear cells were also isolated from leukapheresis using Ficoll-Paque (GE Healthcare, NJ, USA) density gradient media. Peripheral blood mononuclear cells were frozen in 90% FBS and 10% DMSO.

### *In vitro* stimulation of PBMCs

Total PBMCs isolated from adults and old subjects were plated in triplicate at 100 000 cells/well in 96-well U-bottom plates. Cells were suspended in cultured RPMI medium [RPMI with L-glutamine (Corning Cellgro, Manassas, VA, USA) supplemented with 10% FBS and 1× (50U) penicillin–streptomycin (Life Technologies, Carlsbad, CA, USA)]. TLR and RIG-I agonists were added at the following concentrations: LPS-TLR4 (0.5 μg mL^−1^) or CLO97-TLR7/8 (0.5 μg mL^−1^) or poly I:C/Lyovec-RIGI/MDA5 (2 μg mL^−1^) or 5′-pppRNA/Lyovec-RIG-I (400 ng mL^−1^). Optimal concentrations of different TLR agonists were selected based on median production of IL-6 and IFN-α and a ≥ 85% survival rate of PBMCs. All PRR ligands were purchased commercially (InvivoGen, San Diego, CA, USA) except for 5′-pppRNA, which was custom synthesized, as previously described missing reference (Goulet, ML et al, 2013). Peripheral blood mononuclear cells cultured in medium alone were used as a control for LPS and CLO97. Stimulation by poly I:C and 5′-pppRNA required the use of a cationic transfection agent LyoVec (InvivoGen), so medium plus Lyovec alone was used as a control for these two agonists. Peripheral blood mononuclear cells were cultured for 6 and 24 h at 37 °C in a 5% CO_2_-humidified environment.

### Microarray and bioinformatics analyses

Cells were washed twice with cold PBS and lysed with 100 μl of cold RLT buffer (QIAGEN, Frederick, MD, USA) supplemented with 1% beta-mercaptoethanol (BM) (Sigma, St. Louis, MO, USA) and transferred to 1.7-mL tubes with 250 μL of cold RLT/BM buffer and quickly stored at −80 °C. RNA was isolated using a Qiagen's RNeasy Micro Kit followed by DNase I treatment. Quantification was performed using a NanoDrop spectrophotometer (Thermo Scientific, Wilmington, DE, USA), and RNA quality was assessed using Experion automated electrophoresis system (Bio-Rad, Hercules, CA, USA) with a HeLa RNA-positive control and nontemplate negative control. RNA was converted into biotinylated cRNA using the Illumina Total Prep-96 RNA amplification kit (Life Technologies). Biotinylated cRNA was normalized and hybridized to the Illumina Human HT-12V4 Expression BeadChips according to the manufacturer's instruction and quantified using an Illumina iScan System (illumina, San Diego, CA, USA). The data were collected using Illumina GenomeStudio software. Analysis of the microarray output data was conducted using the r statistical language (R Core Team, 2010) and the LIMMA and Combat (batch correction) statistical packages from Bioconductor (Gentleman *et al*., 2004). Microarrays displaying unusually low median intensity, low variability, or low correlation relative to the bulk of the arrays were discarded from the rest of the analysis. Quantile normalization, followed by a log2 transformation using the LIMMA package, was applied to process microarrays. Subsequently, the LIMMA package was used to fit a linear model to each probe and to perform a (moderated) Student's *t-*test on various differences of interest (Smyth *et al*., 2005). In addition, the LIMMA package from Bioconductor was used to identify differentially expressed genes (DEGs) between treated vs. controls (untreated or Lyovec only) or adult vs. old PBMCs. For data mining and functional analyses, DEGs satisfied a stringent false discovery rate (FDR) of 5% with ≥ 1.3 or ≤ −1.3 fold change (FC). Probes that did not map to annotated RefSeq genes and control probes were removed. The expected proportions of false positives (FDR 5%) were estimated from the unadjusted *P* value using the Benjamini and Hochberg method (Benjamini & Hochberg, 1995). An innate gene filter derived from Gene Ontology (GO) was used to highlight innate immunity-related genes within the datasets. The filter was based on innate immune response query (GO: 0045087). All network analysis was performed with ingenuity pathway analysis (IPA: Ingenuity systems). Illumina Probe IDs were imported into IPA and mapped to the gene symbol. Differentially expressed genes (selected based on a nominal *P* value ≤ 0.05 and FC ≥ ± 1.3) that were associated with a canonical pathway in Ingenuity's Knowledge Base were used for pathway analysis. The significance of the association between the dataset and the canonical pathway was measured in two ways: (i) A ratio of the number of genes from the dataset that map to the pathway divided by the total number of genes that map to the canonical pathway was displayed, and (ii) over-representation of Fisher's exact test was used to calculate a *P* value determining the probability that the association between the genes in the dataset and the canonical pathway was explained by chance alone. The pathways were ranked by −log *P* value. Microarray data are available at the NCBI GENE Expression Omnibus (GSE62627).

### Flow cytometry analysis

PBMCs were stained for monocyte subsets using the following cocktail panel: CD19 PE Cy7, CD3 APC Cy7, CD16 AF700, and CD14 PerCp. Dendritic cells were pre-enriched from PBMCs using a pan-DC enrichment kit from Stem Cell Technologies (Vancouver, DC, Canada). DC subsets were stained using the following cocktail panel: CD3 PE Cy5, CD19 PE Cy7, HLA-DR AF700, CD11c PB, CD1c APC Cy7, CD141 FITC, and CD303 APC. Absolute numbers of cell subsets for each subject were determined by dividing individual subset frequencies by total lymphocyte (for monocytes subsets) or white blood cell (for DC subsets) count obtained from Coulter counter (Beckman Coulter, Pasadena, CA, USA). For surface phenotypic analysis, PBMCs were cultured with or without CLO97 for 24 h and stained with CD3 APC-Cy7, CD19 PE Cy7, CD14 PerCp, CD16 AF700, and CD11c PB with one of the following molecules: TLR3 PE, TLR4 PE, TLR7 PE, CD40 PE, CTLA PE, PD-L1 PE, PD-L2 PE, B7-H4 PE, or ICOSL PE. All antibodies were obtained from Biolegend, except for CD3-APC Cy7 (BD Bioscience, Franklin Lakes, NJ, USA), TLR7 PE, and CD14 PerCP (R&D Systems, Minneapolis, MN, USA), CD3 PECy5 (Affymetrix eBioscience, San Diego, CA, USA), and CD141 FITC and CD303 APC (Miltenyi, San Diego, CA, USA). Dead cells were identified using LIVE/DEAD Fixable Aqua Dead Cell Stain Kit for flow cytometry (Life Technologies). Cells were evaluated on a BD LSR II flow cytometer, and frequencies were gated using bd diva software.

### Cytokine and chemokine analysis

Supernatants collected from stimulated PBMCs at 6 and 24 h were analyzed for Th1/Th2/Th17 cytokines (IL-2, IL-4, IL-6, IL-10, TNF, IFN-γ, and IL-17A) and chemokines (CCL2, CCL5, CXCL8, CXCL9, and CXCL10) using BD human cytometric bead array kits (CBA) (BD Biosciences). Samples were acquired on a BD FACSArray and analyzed using fcap array software (BD Biosciences). IFN-α production at 6 and 24 h was measured using an IFN-α ELISA (PBL Interferon Source). Supernatants collected at 24 h were also analyzed using Milliplex MAP magnetic bead assays (Millipore, Billerica, MA, USA). Two human Milliplex premixed panels were used. Panel 1 38-plex: EGF, eotaxin (CCL11), FGF-2, Flt-3 ligand, fractalkine (CX3CL1), G-CSF, GM-CSF, GRO (CXCL1), IFN-α2, IFN-γ, IL-10, IL-12 (p40), IL-12 (p70), IL-13, IL-15, IL-17, IL-1ra, IL-1α, IL-1β, IL-2, IL-3, IL-4, IL-5, IL-6, IL-7, IL-8, IL-9, IP-10 (CXCL10), MCP-1 (CCL2), MCP-3 (CCL7), MDC (CCL22), MIP-1α (CCL3), MIP-1β (CCL4), TGFα, TNF-α, TNF-β, VEGF, and sCD40L. Panel 2 23-plex: (CCL21) 6Ckine, BCA-1 (CXCL13), CTACK (CCL27), ENA-78 (CXCL5), eotaxin-2 (CCL24), eotaxin-3 (CCL26), I-309 (CCL1), IL-16, IL-20, IL-21, IL-23, IL-28A, IL-33, LIF, MCP-2 (CCL8), MCP-4 (CCL13), MIP-1δ (CCL15), SCF, SDF-1A+β (CXCL12), TARC (CCL17), TPO, TRAIL, and TSLP. The manufacturer's protocol was followed. Data were acquired on a Bio-Plex 200 System (using bead regions defined in the Milliplex protocol) and analyzed with the bio-plex manager 6.1 software from Bio-Rad.

### Allogeneic mixed lymphocyte reaction

CD3 T cells were isolated from total PBMCs obtained from healthy adult subjects (pooled two subjects) by negative isolation using a human T-cell enrichment kit (Stem cell). Prior to CD3 enrichment, PBMCs were labeled with carboxyfluorescein diacetate succinimidyl ester (CFSE; Life Technologies) for subsequent assessment of T-cell proliferation. For agonist-stimulated PBMCs, CD3 T cells were removed from PBMCs by positive selection using a human T-cell enrichment kit (Stem cells). Harvested PBMCs (minus CD3 T cells) were adjusted to a concentration of 2x10^6^ cells mL^−1^ with cultured RPMI medium and cultured with 0.1 μg mL^−1^ LPS or 1 μg mL^−1^ CLO97 or without stimuli in a 37 °C, 5% CO_2_ atmosphere for 20 hrs. Agonist-stimulated PBMCs (minus CD3 T cells) from adult and old subjects were washed three times with cultured RPMI medium and co-incubated 1:1 with T cells from young subjects in a 37 °C, 5% CO_2_ atmosphere for 5 days. For the gating of CFSE^low^CD3+ T cells, frequencies with a gating of > 20 events were considered significant. Peripheral blood mononuclear cells used for these experiments were obtained from leukapheresis of healthy adult and old subjects.

### Statistical methods

For DC/monocyte, TLR, CD40, and MLR analysis, *P* values were determined by two-sided nonparametric Mann–Whitney *U*-test, which ranks the data rather than their raw values to calculate the statistical significance and is an alternative to the t-test, when the assumption of normality is not satisfied or could not be tested in the case of small sample sizes. For cytokine production and surface receptor upregulation analysis, significant differences between multiple comparisons (LPS or CLO97-treated vs. untreated PBMCs, poly I:C/Lyovec or 5′-pppRNA/Lyovec-treated vs. Lyovec-only treated PBMCs, and adult vs. old PBMCs) were determined by two-way ANOVA test followed by Sidak correction for multiple comparisons. A *P* value < 0.05 was considered significant. Data were analyzed and figures generated using graphpad prism 6 software (graphpad software, La Jolla, CA, USA).

## Supporting Information

**Fig. S1** Absolute number and frequency of monocyte and dendritic cell subsets is unchanged with age. Representative dot plots depicting the gating scheme for (A) CD14^dim^CD16^+^, CD14^+^CD16^+^ and CD14^+^CD16^−^ monocyte subsets and (B) mDC1 (CD11c^+^CD1c^+^CD141^−^CD303^−^), pDCs (CD11c^−^CD1c^−^ CD141^−^CD303^+^) and mDC2 (CD11c^lo^CD1c^−^CD141^+^CD303^−^) DC subsets. Graphs depict the absolute numbers and frequencies for monocytes (C and E) and DCs (D and F). Individual values along with mean ± SEM are shown (*n *=* *8/group except for mDC2% *n *=* *5/group). Adults were compared to old subjects by Mann-Whitney *U* test; and *P* values are given if the differences are statistically significant. **Fig. S2** Expression of TLR3, TLR4 and TLR9 on monocytes was unchanged with age. Peripheral blood mononuclear cells isolated from adult and old subjects were stained for CD14^dim^CD16^+^, CD14^+^CD16^+^ and CD14^+^CD16^−^ monocyte subsets and either TLR 3 PE, TLR 4 PE or TLR 7 PE. Frequency values along with mean ± SEM are shown (*n *=* *7/group). Adults were compared to old subjects by Mann-Whitney *U* test; and *P* values are given if the differences are statistically significant. **Fig. S3** Frequency of CD40+ B cells was reduced in PBMCs from old subjects compared to adults. Peripheral blood mononuclear cells isolated from adult and old subjects were stained for CD19+ CD40+ B cells. Frequencies (within total B cells) along with mean ± SEM are shown (*n *=* *6/group). Adults were compared to old subjects by Mann-Whitney *U* test. **Table s1** Transcriptional profiles for agonist-treated PBMCs from adults compared to negative controls. The table lists DEGs resulting from contrasting agonist-stimulated PBMCs from adults (*n *=* *8) to negative controls. Differentially expressed genes were selected based on FC ≥ 1.3 or ≤ −1.3 and FDR 5%. For poly I:C/Lyovec 6 h, genes were selected based on nominal *P* value < 0.05 and FC ≥ 1.3 or ≤ −1.3 due to FDR 5% resulting in zero genes. **Table s2** Transcriptional profiles for agonist-treated PBMCs from old subjects compared to negative controls. The table lists DEGs resulting from contrasting agonist-stimulated PBMCs from old subjects (*n *=* *8) to negative controls. Differentially expressed genes were selected based on FC ≥ 1.3 or ≤ −1.3 and FDR 5%. For poly I:C/Lyovec 6 h, genes were selected based on nominal *P* value < 0.05 and FC ≥ 1.3 or ≤ −1.3 due to FDR 5% resulting in zero genes. **Table S3** Venn diagram analysis contrasting adult to old PBMCs stimulated with PRR agonists. The table lists unique and common DEGs resulting from Venn diagram analysis comparing cells from adult and old subjects (*n *=* *8/group) stimulated with agonists (both age groups normalized to their respective negative controls). Differentially expressed genes were selected based on FC ≥ 1.3 or ≤ −1.3 and FDR 5%. **Table S4** IPA pathway analysis contrasting stimulated to negative control PBMCs. Table lists *P* values, pathway enrichment scores, and enriched molecules resulting from IPA pathway analysis contrasting stimulated to untreated (LPS and CLO97) or Lyovec only (5′-pppRNA/Lyovec and poly I:C/Lyovec) PBMCs for adult and old individuals (*n *=* *8/group). **Table S5** Specific cell type meta analysis contrasting adults to old subjects. Table lists normalized enrichment scores (NES), *P* values, and enriched molecules resulting from specific cell-type meta analysis contrasting adults to old subjects (*n *=* *8/group) stimulated with agonists. Positive (+) NES represent upregulation in adults, whereas a negative (−) NES represent upregulation in old subjects. **Table S6** Concentrations (pg mL^−1^) of cytokines and chemokines induced by agonists stimulation of PBMCs. Table lists cytokine and chemokine concentrations (mean ± SEM) elicited by no treatment, LPS, CLO97, and Lyovec-treated only, 5′-pppRNA/Lyovec, and poly I:C/Lyovec (*n *=* *8/group). 6 h concentration levels measured by ELISA and CBA, whereas 24 h levels measured by Milliplex bead assays. Assay sensitivities listed in table were taken from the manufacturer's manual (LOD = below limited of detection). Statistical significance between untreated vs. treated and adult vs. old subjects was determined by two-way ANOVA followed by Sidak correction for multiple comparisons. (*) *P* < 0.05, (**) *P* < 0.01, (***) *P* < 0.00, and (****) *P* < 0.0001. **Table S7** Description of adult and old cohorts. Table depicts total number of subjects used in this study.
