# Supplementary material for: Global analyses revealed age-related alterations in innate immune responses after stimulation of pathogen recognition receptors
Source: Aging Cell. 2015 Feb 27;14(3):421–32. doi: 10.1111/acel.12320 (PMC4406671; doi:10.1111/acel.12320)
Supplement: Supplementary file 1 [file acel0014-0421-sd1.zip › SuppInform Table 4 IPA pathways_revised.pdf]

Adult LPS 6 h vs no treatement

Ingenuity Canonical Pathways	-log(p-value)	p-value	Pathway enrichment score	Pathway enrichment score %	Molecules
Activation of IRF by Cytosolic Pattern Recognition Receptors	5.91E00	1.23027E-06	3.33E-01	33.3	DHX58,IL10,CREBBP,ZBP1,IRF9,IL6,NFKB2,NFKB1,ADAR,ISG15,IFIH1,TANK,IRF7,RIPK1,NFKBIA,LTA,IFNA7,DDX58,STAT2,STAT1,NFKBIB,IFIT2,TNF,IFNAR1
Communication between Innate and Adaptive Immune Cells	5.06E00	8.70964E-06	2.64E-01	26.4	B2M,IL1A,TLR8,CD83,CCL5,IGHG1,IL6,CD8A,CCL3,HLA-F,CXCL10,IL36G,HLA-A,HLA-B,TLR7,HLA-G,IFNG (includes EG:15978),IL8,IL10,IL18 (includes EG:16173),CCL4,TLR5,HLA-E,IL1RN,CCL3L1/CCL3L3,IL1B,CSF2,TNF,CCR7
Crosstalk between Dendritic Cells and Natural Killer Cells	6.97E00	1.07152E-07	3.37E-01	33.7	KLRD1,ACTA2,LTB,KIR3DL2,TNFSF10,CD83,IL6,NFKB1,IL2RB,FAS,HLA-F,PRF1,MICB,HLA-A,HLA-B,TLR7,LTBR,TNFRSF1B,HLA-G,CAMK2B,IFNG (includes EG:15978),KIR2DL3 (includes others),CD69,NFKB2,CSF2RB,IL18 (includes EG:16173),HLA-E,LTA,CSF2,TNF,CCR7,PVRL2
IL-10 Signaling	5.23E00	5.88844E-06	3.08E-01	30.8	CCR1,MAP2K6,IL18RAP,IL4R,IL1A,JAK1 (includes EG:16451),IL10,MAP4K4,IL6,NFKB2,IL1R1,NFKB1,FCGR2B,MAPK11,HMOX1,IL36G,IL18 (includes EG:16173),NFKBIA,IL1RN,CD14,IL1B,MAP2K3,NFKBIB,TNF
IL-15 Production	1.17E00	0.067608298	2.26E-01	22.6	PTK2 (includes EG:14083),JAK1 (includes EG:16451),NFKB2,IL6,STAT1,NFKB1,IRF1 (includes EG:16362)
IL-6 Signaling	3.09E00	0.000812831	2.34E-01	23.4	MAP2K6,IL18RAP,SOCS1,IL1A,PIK3CA,PIK3R1,MAP4K4,IL6,NFKB1,MAPK11,VEGFA,IL36G,NFKBIA,PIK3CG,TNFRSF1B,NFKBIB,ATM,MCL1,IL8,TNFRSF1A,IL1R1,NFKB2,IL18 (includes EG:16173),IL1RN,CD14,IL1B,MAP2K3,TNFAIP6,TNF
Interferon Signaling	7.17E00	6.76083E-08	5E-01	50	IFNG (includes EG:15978),IFIT3,SOCS1,OAS1,JAK1 (includes EG:16451),IRF9,MX1,IFI35,BAX,PSMB8,TAP1,IRF1 (includes EG:16362),MED14,IFIT1,IFITM1,STAT2,STAT1,IFNAR1
JAK/Stat Signaling	3.33E00	0.000467735	2.86E-01	28.6	STAT5A,STAT6 (includes EG:20852),SOCS1,PTPN6,PIK3CA,JAK1 (includes EG:16451),PIK3R1,SOCS4,IL6,STAT4,BCL2L1,MTOR,PIK3CG,CISH,PTPN1,STAT2,STAT5B,STAT1,SOCS7,ATM
PPAR Signaling	3.47E00	0.000338844	2.45E-01	24.5	IL18RAP,IL1A,MAP4K4,NFKB1,IL36G,NFKBIA,HSP90AB1,NFKBIB,TNFRSF1B,STAT5B,CITED2,PPARG,STAT5A,TNFRSF1A,CREBBP,IL1R1,NFKB2,IL18 (includes EG:16173),IL1RN,HSP90AA1,IL1B,NCOR2,PTGS2,RXRA,TNF,PPARGC1A

Role of Cytokines in Mediating Communication between Immune Cells	2.22E00	0.006025596	2.73E-01	27.3	IL8,IFNG (includes EG:15978),IL1A,IL10,IL32,IL6,CSF3,IL36G,IL18 (includes EG:16173),IL1RN,IFNA7,IL1B,CSF2,IL23A,TNF
Role of IL-17A in Psoriasis	2.32E00	0.004786301	4.62E-01	46.2	S100A7,IL8,CXCL3,CXCL1,CCL20,S100A8
Role of IL-17F in Allergic Inflammatory Airway Diseases	2.41E00	0.003890451	2.83E-01	28.3	IL8,CXCL1,NFKB2,IL6,NFKB1,CXCL10,TRAF3IP2,CCL4,CREB1,ATF4,IL1B,RPS6KA5,CSF2
Role of JAK family kinases in IL-6-type Cytokine Signaling	1.33E00	0.046773514	2.59E-01	25.9	SOCS1,STAT5A,JAK1 (includes EG:16451),IL6,STAT1,STAT5B,MAPK11
Role of JAK1, JAK2 and TYK2 in Interferon Signaling	2.5E00	0.003162278	3.33E-01	33.3	SOCS1,IFNG (includes EG:15978),PTPN6,JAK1 (includes EG:16451),STAT2,NFKB2,STAT1,NFKB1,IFNAR1
Role of Pattern Recognition Receptors in Recognition of Bacteria and Viruses	4.22E00	6.0256E-05	2.64E-01	26.4	PIK3CA,PIK3R1,TLR8,CCL5,IL6,NFKB1,IFIH1,C5AR1,TICAM1,PIK3CG,CREB1,TLR7,RIPK2,ATM,OAS1,NLRP3,OAS2,IL10,NFKB2,OAS3,IRF7,NOD2,TLR5,DDX58,IL1B,EIF2AK2,TNF,C3AR1
Role of RIG1-like Receptors in Antiviral Innate Immunity	2.31E00	0.004897788	2.65E-01	26.5	DHX58,CREBBP,NFKB2,NFKB1,TANK,IFIH1,IRF7,RIPK1,NFKBIA,DDX58,IFNA7,NFKBIB,TRIM25
TREM1 Signaling	4.17E00	6.76083E-05	2.82E-01	28.2	ITGB1,STAT5A,IL8,IL10,TLR8,CD83,IL6,NFKB2,NFKB1,FCGR2B,CCL3,CXCL3,NOD2,IL18 (includes EG:16173),TLR5,TLR7,IL1B,STAT5B,CSF2,TNF

Adult LPS 24 h vs no treatment

Ingenuity Canonical Pathways	-log(p-value)	p-value	Pathway enrichment score	Pathway enrichment score %	Molecules
Activation of IRF by Cytosolic Pattern Recognition Receptors	1.47E00	0.033884416	1.39E-01	13.9	IRF7,RIPK1,IL10,NFKBIE,MAPK8,IKBKE,IRF9,NFKB2,IL6,STAT1
Communication between Innate and Adaptive Immune Cells	2.87E00	0.001348963	1.55E-01	15.5	TLR1,IFNG (includes EG:15978),IL8,IL1A,IL10,TLR8,IL6,CCL3,HLA-F,IL36G,IL18 (includes EG:16173),CD86,IL1B,CCL3L1/CCL3L3,TNFRSF13B,CSF2,CCR7
Crosstalk between Dendritic Cells and Natural Killer Cells	6.82E00	1.51356E-07	2.53E-01	25.3	IFNG (includes EG:15978),KIR3DL1,KIR2DL3 (includes others),IL3RA,TYROBP,KLRD1,ACTB,CD69,LTB,TLN1,IL6,NFKB2,KIR2DL4/LOC100287534,ACTG1,IL2RB,HLA-F,IL18 (includes EG:16173),KIR2DL5A,FSCN1,CD86,LTBR,CSF2,CCR7,PVRL2
IL-10 Signaling	7.15E00	7.07946E-08	2.69E-01	26.9	CCR1,IL1A,MAPK1,IL10,FCGR2A,NFKBIE,BLVRB,MAPK8,IKBKE,IL6,STAT3,NFKB2,FCGR2B,IL1R2,FOS,IL18 (includes EG:16173),IL36G,CD14,IL1B,MAP2K3,TAB1
IL-15 Production	1.68E00	0.020892961	1.94E-01	19.4	MAP3K11,NFKB2,IL6,STAT1,PRKCZ,IRF1 (includes EG:16362)
IL-6 Signaling	5.13E00	7.4131E-06	2.02E-01	20.2	SOCS1,IL1A,MAPK1,NFKBIE,PIK3R1,MAPK3,IL6,IL1R2,IL36G,TAB1,ATM,IL8,RRAS,GRB2,TNFRSF1A,MAPK8,IKBKE,STAT3,NFKB2,FOS,IL18 (includes EG:16173),CD14,IL1B,MAP2K3,TNFAIP6
Interferon Signaling	5.84E00	1.44544E-06	3.61E-01	36.1	SOCS1,IFNG (includes EG:15978),IFNGR2,IFNGR1,IFI35,IRF9,PSMB8,BAX,TAP1,MED14,IRF1 (includes EG:16362),IFITM1,STAT1
JAK/Stat Signaling	3.51E00	0.00030903	2.14E-01	21.4	STAT5A,SOCS1,PTPN6,MAPK1,GRB2,RRAS,MAPK3,PIK3R1,IL6,STAT3,STAT4,FOS,PTPN1,STAT1,ATM
PPAR Signaling	5.11E00	7.76247E-06	2.08E-01	20.8	STAT5A,SRA1,IL1A,MAPK1,TNFRSF1A,GRB2,RRAS,NFKBIE,MAPK3,NR1H3,IKBKE,NFKB2,PDGFC,IL1R2,FOS,IL18 (includes EG:16173),IL36G,HSP90AA1,IL1B,PTGS2,RXRA,TAB1
Role of Cytokines in Mediating Communication between Immune Cells	2.81E00	0.001548817	2.18E-01	21.8	IL8,IFNG (includes EG:15978),IL36G,IL1A,IL18 (includes EG:16173),IL10,IL32,IL1B,IL6,IL23A,CSF2,IL24
Role of IL-17A in Psoriasis	4.54E00	2.88403E-05	5.38E-01	53.8	DEFB4A/DEFB4B,IL8,CXCL1,CCL20,S100A8,IL17RA,CXCL6

Role of JAK family kinases in IL-6-type Cytokine Signaling	3.18E00	0.000660693	2.96E-01	29.6	SOCS1,STAT5A,MAPK1,MAPK3,MAPK8,STAT3,IL6,STAT1
Role of JAK1, JAK2 and TYK2 in Interferon Signaling	3.32E00	0.00047863	2.96E-01	29.6	SOCS1,IFNG (includes EG:15978),PTPN6,IFNGR2,IFNGR1,STAT3,NFKB2,STAT1
Role of Pattern Recognition Receptors in Recognition of Bacteria and Viruses	4.51E00	3.0903E-05	1.98E-01	19.8	TLR1,MAPK1,IL10,PIK3R1,MAPK3,TLR8,MAPK8,IL6,NFKB2,PRKCZ,IRF7,NOD2,C5AR1,TICAM1,SYK,PRKCD,CREB1,IL1B,C3AR1,RIPK2,ATM
Role of RIG1-like Receptors in Antiviral Innate Immunity	5.24E-01	0.299226464	1.02E-01	10.2	IRF7,RIPK1,NFKBIE,IKBKE,NFKB2
TREM1 Signaling	1.09E01	1.25893E-11	3.38E-01	33.8	DEFB4A/DEFB4B,TLR1,STAT5A,IL8,MAPK1,TYROBP,GRB2,IL10,MAPK3,TLR8,ITGA5,STAT3,IL6,NFKB2,CCL3,FCGR2B,NOD2,IL18 (includes EG:16173),CCL2,CCL7,CD86,IL1B,CSF2,ITGAX

Adult CLO97 6 h vs no treatment

Ingenuity Canonical Pathways	-log(p-value)	p-value	Pathway enrichment score	Pathway enrichment score %	Molecules
Activation of IRF by Cytosolic Pattern Recognition Receptors	7.05E00	8.91251E-08	3.61E-01	36.1	NFKBIE,ZBP1,IL6,NFKB1,IFIH1,TANK,NFKBIA,IFNA7,NFKBIB,STAT1,DHX58,IL10,IRF9,TBK1,NFKB2,ADAR,ISG15,IRF7,RIPK1,CD40,LTA,DDX58,STAT2,IFIT2,TNF,IFNAR1
Communication between Innate and Adaptive Immune Cells	6.46E00	3.46737E-07	2.91E-01	29.1	B2M,IL1A,TLR8,CD83,CCL5,IL6,CCL3,HLA-F,CXCL10,IL36G,HLA-A,HLA-DRA,HLA-B,TLR7,HLA-G,CCL15,TNFSF13B,IFNG (includes EG:15978),IL8,IL10,IL15 (includes EG:16168),IL18 (includes EG:16173),CCL4,CD40,TLR5,IL1RN,HLA-E,CCL3L1/CCL3L3,IL1B,CSF2,TNF,CCR7
Crosstalk between Dendritic Cells and Natural Killer Cells	1.12E01	6.30957E-12	4.11E-01	41.1	IL15RA,KLRD1,ACTA2,KIR3DL2,TNFSF10,CD83,IL6,NFKB1,IL2RB,FAS,HLA-F,PRF1,HLA-A,MICB,HLA-DRA,CD226,HLA-B,TLR7,TNFRSF1B,HLA-G,MICA,FASLG,CAMK2B,IFNG (includes EG:15978),KIR2DL3 (includes others),CD69,ACTB,IL15 (includes EG:16168),NFKB2,ACTG1,CSF2RB,IL18 (includes EG:16173),CD40,HLA-E,ICAM3,LTA,CSF2,TNF,CCR7
IL-10 Signaling	5.13E00	7.4131E-06	3.08E-01	30.8	MAP2K6,IL18RAP,SOCS3,IL1A,IL10,NFKBIE,MAP4K4,IL6,NFKB2,STAT3,NFKB1,FCGR2B,MAPK11,HMOX1,IL36G,IL18 (includes EG:16173),NFKBIA,IL1RN,IL10RA,CD14,IL1B,MAP2K3,NFKBIB,TNF
IL-15 Production	7.86E-01	0.163681652	1.94E-01	19.4	IL15 (includes EG:16168),NFKB2,IL6,STAT1,NFKB1,IRF1 (includes EG:16362)
IL-6 Signaling	3E00	0.001	2.34E-01	23.4	MAP2K6,SOCS3,IL18RAP,SOCS1,IL1A,PIK3CA,NFKBIE,SRF,MAP4K4,IL6,NFKB1,MAPK11,VEGFA,IL36G,NFKBIA,AKT1,TNFRSF1B,NFKBIB,MCL1,IL8,STAT3,NFKB2,IL18 (includes EG:16173),IL1RN,CD14,IL1B,MAP2K3,TNFAIP6,TNF
Interferon Signaling	6.25E00	5.62341E-07	4.72E-01	47.2	IFNG (includes EG:15978),IFIT3,SOCS1,OAS1,IRF9,MX1,IFI35,BAX,PSMB8,TAP1,IRF1 (includes EG:16362),MED14,IFIT1,IFITM1,STAT2,STAT1,IFNAR1
JAK/Stat Signaling	2.05E00	0.008912509	2.43E-01	24.3	STAT5A,STAT6 (includes EG:20852),SOCS1,SOCS3,PTPN6,PIK3CA,SOCS4,IL6,STAT3,STAT4,BCL2L1,AKT1,CDKN1A,PTPN1,STAT2,STAT5B,STAT1
PPAR Signaling	2.31E00	0.004897788	2.17E-01	21.7	PPARG,STAT5A,IL18RAP,IL1A,NFKBIE,MAP4K4,NFKB2,NFKB1,IL18 (includes EG:16173),HSP90B1,IL36G,NFKBIA,HSP90AB1,IL1RN,HSP90AA1,IL1B,NCOR2,PTGS2,STAT5B,TNFRSF1B,NFKBIB,TNF,PPARGC1A
Role of Cytokines in Mediating Communication between Immune Cells	3.04	0.000912011	0.309	30.9	IFNG (includes EG:15978),IL8,IL1A,IL10,IL32,IL15 (includes EG:16168),IL27,IL6,CSF3,IL36G,IL18 (includes EG:16173),IL1RN,IFNA7,IL1B,CSF2,IL23A,TNF

Role of IL-17A in Psoriasis	5.73E-01	0.267300641	2.31E-01	23.1	S100A7,IL8,CCL20
Role of IL-17F in Allergic Inflammatory Airway Diseases	2.84E00	0.00144544	3.04E-01	30.4	IL8,NFKB2,IL6,NFKB1,CXCL10,TRAF3IP2,CCL4,CCL2,CCL7,CREB1,ATF4,IL1B,RPS6KA5,CSF2
Role of JAK family kinases in IL-6-type Cytokine Signaling	2.32E00	0.004786301	3.33E-01	33.3	SOCS1,SOCS3,STAT5A,OSM,STAT3,IL6,STAT1,STAT5B,MAPK11
Role of JAK1, JAK2 and TYK2 in Interferon Signaling	2.46E00	0.003467369	3.33E-01	33.3	SOCS1,IFNG (includes EG:15978),PTPN6,STAT2,STAT3,NFKB2,STAT1,NFKB1,IFNAR1
Role of Pattern Recognition Receptors in Recognition of Bacteria and Viruses	3.7E00	0.000199526	2.55E-01	25.5	PIK3CA,TLR8,IL6,CCL5,NFKB1,IFIH1,C5AR1,TICAM1,CREB1,TLR7,CASP1,RIPK2,OAS1,PRKCQ,NLRP3,OAS2,IL10,MYD88,NFKB2,OAS3,IRF7,TLR5,PRKCD,DDX58,IL1B,EIF2AK2,TNF
Role of RIG1-like Receptors in Antiviral Innate Immunity	2.73E00	0.001862087	2.86E-01	28.6	DHX58,NFKBIE,TBK1,NFKB2,NFKB1,TANK,IFIH1,IRF7,NFKBIA,RIPK1,DDX58,IFNA7,NFKBIB,TRIM25
TREM1 Signaling	7.76E00	1.7378E-08	3.66E-01	36.6	ICAM1,TLR8,CD83,IL6,NFKB1,FCGR2B,CCL3,AKT1,CCL2,TLR7,CASP1,STAT5B,ITGB1,IL8,STAT5A,IL10,MYD88,STAT3,NFKB2,IL18 (includes EG:16173),TLR5,CCL7,CD40,IL1B,CSF2,TNF

Adult CLO97 24 h vs no treatment

Ingenuity Canonical Pathways	-log(p-value)	p-value	Pathway enrichment score	Pathway enrichment score %	Molecules
Activation of IRF by Cytosolic Pattern Recognition Receptors	7.04E00	9.12011E-08	2.92E-01	29.2	DHX58,IL10,ZBP1,MAPK8,TBK1,IRF9,IL6,NFKB2,ADAR,IFNA1/IFNA13,ISG15,TANK,IFIH1,IRF7,NFKBIA,CD40,LTA,STAT2,STAT1,IFIT2,TNF
Communication between Innate and Adaptive Immune Cells	5.18E00	6.60693E-06	2.09E-01	20.9	IFNG (includes EG:15978),IL8,IL10,IL15 (includes EG:16168),CD83,IL6,CCL3,IFNA1/IFNA13,HLA-F,CXCL10,TLR10,IL36G,CD40,HLA-E,IL1RN,CCL3L1/CCL3L3,CD86,IL1B,CSF2,TNFRSF13B,TNF,CCR7,TNFSF13B
Crosstalk between Dendritic Cells and Natural Killer Cells	8.53E00	2.95121E-09	2.95E-01	29.5	KIR3DL1,IL15RA,TNFSF10,CD83,IL6,IL2RB,FAS,HLA-F,PRF1,MICB,KIR2DL5A,TNFRSF1B,IFNG (includes EG:15978),CD69,ACTB,IL15 (includes EG:16168),NFKB2,KIR2DL4/LOC100287534,IFNA1/IFNA13,ACTG1,CD40,HLA-E,LTA,FSCN1,CD86,CSF2,TNF,CCR7
IL-10 Signaling	4.55E00	2.81838E-05	2.31E-01	23.1	IL18RAP,SOCS3,IL10,FCGR2A,BLVRB,MAPK8,IL6,NFKB2,FCGR2B,IL1R2,FOS,IL36G,NFKBIA,IL1RN,CD14,IL1B,TNF,TAB1
IL-15 Production	2.03E00	0.009332543	2.26E-01	22.6	IL15 (includes EG:16168),NFKB2,IL6,STAT1,IFNA1/IFNA13,PRKCZ,IRF1 (includes EG:16362)
IL-6 Signaling	4.38E00	4.16869E-05	2.02E-01	20.2	SOCS3,SOCS1,IL18RAP,MAPK3,SRF,IL6,IL1R2,IL36G,NFKBIA,TNFRSF1B,TAB1,MCL1,IL8,PIK3C2B,RRAS,GRB2,MAPK8,NFKB2,FOS,TRAF2,IL1RN,CD14,IL1B,TNFAIP6,TNF
Interferon Signaling	9.99E00	1.02329E-10	5E-01	50	IFNG (includes EG:15978),IFIT3,SOCS1,OAS1,IRF9,MX1,IFNGR1,IFI35,BAX,PSMB8,IFNA1/IFNA13,TAP1,MED14,IRF1 (includes EG:16362),IFIT1,IFITM1,STAT2,STAT1
JAK/Stat Signaling	2.14E00	0.00724436	1.86E-01	18.6	SOCS1,SOCS3,PIK3C2B,STAT5A,GRB2,RRAS,MAPK3,IL6,FOS,CDKN1A,PTPN1,STAT2,STAT1
PPAR Signaling	6.02E00	9.54993E-07	2.36E-01	23.6	IL18RAP,MAPK3,PDGFC,IL1R2,IL36G,NFKBIA,HSP90AB1,TNFRSF1B,TAB1,STAT5A,SRA1,PPARD,RRAS,GRB2,NR1H3,NFKB2,FOS,TRAF2,IL1RN,HSP90AA1,IL1B,NCOR2,PTGS2,RXRA,TNF
Role of Cytokines in Mediating Communication between Immune Cells	2.94E00	0.001148154	2.36E-01	23.6	IL8,IFNG (includes EG:15978),IL10,IL15 (includes EG:16168),IL6,IFNA1/IFNA13,IL24,IL36G,IL1RN,IL1B,IL23A,CSF2,TNF
Role of IL-17A in Psoriasis	1.59E00	0.025703958	3.08E-01	30.8	DEFB4A/DEFB4B,IL8,CXCL1,CXCL6
Role of IL-17F in Allergic Inflammatory Airway Diseases	3.45E00	0.000354813	2.61E-01	26.1	CXCL10,IL8,TRAF3IP2,CCL2,CCL7,MAPK3,CXCL1,IL1B,NFKB2,IL6,CSF2,CXCL6
Role of JAK family kinases in IL-6-type Cytokine Signaling	2.23E00	0.005888437	2.59E-01	25.9	SOCS1,SOCS3,STAT5A,MAPK3,MAPK8,IL6,STAT1
Role of JAK1, JAK2 and TYK2 in Interferon Signaling	2.33E00	0.004677351	2.59E-01	25.9	SOCS1,IFNG (includes EG:15978),IFNGR1,STAT2,NFKB2,STAT1,IFNA1/IFNA13
Role of Pattern Recognition Receptors in Recognition of Bacteria and Viruses	3.42E00	0.000380189	1.89E-01	18.9	PIK3C2B,OAS1,OAS2,IL10,MAPK3,MAPK8,IL6,NFKB2,OAS3,IFNA1/IFNA13,PRKCZ,IFIH1,IRF7,C5AR1,CASP1,IL1B,EIF2AK2,TNF,C3AR1,RIPK2
Role of RIG1-like Receptors in Antiviral Innate Immunity	2.25E00	0.005623413	2.04E-01	20.4	DHX58,TANK,IFIH1,TRAF2,IRF7,NFKBIA,TBK1,NFKB2,IFNA1/IFNA13,TRIM25
TREM1 Signaling	8.38E00	4.16869E-09	3.1E-01	31	DEFB4A/DEFB4B,STAT5A,IL8,ICAM1,GRB2,IL10,MAPK3,CD83,IL6,NFKB2,FCGR2B,CCL3,TLR10,CCL2,CCL7,CD40,CASP1,CD86,IL1B,CSF2,TNF,CASP5

Adult 5'pppRNA 6 h vs Lyovec

Ingenuity Canonical Pathways	-log(p-value)	p-value	Pathway enrichment score	Pathway enrichment score %	Molecules
Activation of IRF by Cytosolic Pattern Recognition Receptors	5.4E00	3.98107E-06	1.81E-01	18.1	DHX58,IFNB1 (includes EG:15977),NFKB2,IL6,ISG15,IFIH1,IRF7,RIPK1,DDX58,STAT2,STAT1,IFIT2,TNF
Communication between Innate and Adaptive Immune Cells	2.53E00	0.002951209	1E-01	10	CXCL10,IL1A,IL1RN,IFNB1 (includes EG:15977),CCL3L1/CCL3L3,CD86,IL6,CD8A,CSF2,TNF,TNFSF13B
Crosstalk between Dendritic Cells and Natural Killer Cells	2.16E00	0.00691831	1.05E-01	10.5	KIR2DL3 (includes others),ACTB,IFNB1 (includes EG:15977),CD86,TNFSF10,NFKB2,IL6,CSF2,TNF,ACTG1
IL-10 Signaling	3.03E00	0.000933254	1.28E-01	12.8	FOS,IL1A,JAK1 (includes EG:16451),IL1RN,IL10RB,MAP2K3,STAT3,NFKB2,IL6,TNF
IL-15 Production	2.18E00	0.006606934	1.61E-01	16.1	JAK1 (includes EG:16451),IFNB1 (includes EG:15977),NFKB2,IL6,STAT1
IL-6 Signaling	5.33E00	4.67735E-06	1.45E-01	14.5	SOCS1,IL1A,GRB2,PIK3R1,PIK3R5,IL6,NFKB2,STAT3,VEGFA,FOS,IL1RN,CSNK2A1,PIK3CB,MAP2K3,TNFAIP6,TNF,ATM,MCL1
Interferon Signaling	1.25E01	3.16228E-13	4.44E-01	44.4	IFIT3,SOCS1,OAS1,JAK1 (includes EG:16451),PTPN2,IFNB1 (includes EG:15977),MX1,IFNGR1,IFI35,PSMB8,IFNAR2,TAP1,IFIT1,IFITM1,STAT2,STAT1
JAK/Stat Signaling	5.09E00	8.12831E-06	1.86E-01	18.6	SOCS1,JAK1 (includes EG:16451),GRB2,PIK3R1,PIK3R5,SOCS4,STAT3,IL6,FOS,STAT2,PIK3CB,STAT1,ATM
PPAR Signaling	1.55E00	0.028183829	8.49E-02	8.49	FOS,IL1A,GRB2,IL1RN,PTGS2,NFKB2,TNF,SCAND1,EP300
Role of Cytokines in Mediating Communication between Immune Cells	1.93E00	0.011748976	1.27E-01	12.7	IL1A,IL1RN,IFNB1 (includes EG:15977),IL6,CSF2,IL24,TNF
Role of IL-17A in Psoriasis	3.43E-01	0.453941617	7.69E-02	7.69	CXCL5
Role of IL-17F in Allergic Inflammatory Airway Diseases	1.9E00	0.012589254	1.3E-01	13	CXCL10,RPS6KA3,CXCL5,NFKB2,IL6,CSF2
Role of JAK family kinases in IL-6-type Cytokine Signaling	2.32E00	0.004786301	1.85E-01	18.5	SOCS1,JAK1 (includes EG:16451),STAT3,IL6,STAT1
Role of JAK1, JAK2 and TYK2 in Interferon Signaling	7.42E00	3.80189E-08	3.7E-01	37	SOCS1,JAK1 (includes EG:16451),PTPN2,IFNB1 (includes EG:15977),IFNGR1,STAT2,STAT3,NFKB2,STAT1,IFNAR2
Role of Pattern Recognition Receptors in Recognition of Bacteria and Viruses	6.58E00	2.63027E-07	1.7E-01	17	OAS1,NLRP3,OAS2,PIK3R1,IFNB1 (includes EG:15977),PIK3R5,IL6,NFKB2,OAS3,IFIH1,IRF7,DDX58,PIK3CB,EIF2AK2,TNF,RIPK2,ATM,PRKCB
Role of RIG1-like Receptors in Antiviral Innate Immunity	3.88E00	0.000131826	1.84E-01	18.4	DHX58,IFIH1,IRF7,RIPK1,DDX58,IFNB1 (includes EG:15977),NFKB2,TRIM25,EP300
TREM1 Signaling	4.23E00	5.88844E-05	1.55E-01	15.5	ITGB1,TREM1,ICAM1,GRB2,LAT2,CD86,STAT3,NFKB2,IL6,CSF2,TNF

Adult 5'pppRNA 24 h vs Lyovec

Ingenuity Canonical Pathways	-log(p-value)	p-value	Pathway enrichment score	Pathway enrichment score %	Molecules
Activation of IRF by Cytosolic Pattern Recognition Receptors	8.25E00	5.62341E-09	2.78E-01	27.8	DHX58,IFNA8,ZBP1,IFNB1 (includes EG:15977),IRF9,IFNA16,ADAR,IFNA1/IFNA13,IFNA14,ISG15,IFIH1,IRF7,RIPK1,CD40,LTA,DDX58,IFNA2,STAT2,STAT1,IFIT2
Communication between Innate and Adaptive Immune Cells	4.2E00	6.30957E-05	1.64E-01	16.4	IFNG (includes EG:15978),IL1A,IL15 (includes EG:16168),IFNB1 (includes EG:15977),CD8A,IFNA1/IFNA13,HLA-F,CXCL10,CD40,CD80 (includes EG:12519),IL1RN,TLR7,CD86,CCL3L1/CCL3L3,IL1B,TNFRSF13B,TNFSF13B,CCR7
Crosstalk between Dendritic Cells and Natural Killer Cells	5.55E00	2.81838E-06	2.11E-01	21.1	IFNG (includes EG:15978),CD69,IL15 (includes EG:16168),ACTA2,IFNB1 (includes EG:15977),TNFSF10,TLN1,IFNA1/IFNA13,ACTG1,HLA-F,PRF1,CD80 (includes EG:12519),CD40,MICB,LTA,FSCN1,TLR7,CD86,CCR7,PVRL2
IL-10 Signaling	3.63E00	0.000234423	1.79E-01	17.9	CCR1,IL18RAP,IL1A,IL4R,JAK1 (includes EG:16451),MAPK1,BLVRA,FCGR2A,FCGR2B,FOS,IL1RN,IL10RA,CD14,IL1B
IL-15 Production	2E00	0.01	1.94E-01	19.4	JAK1 (includes EG:16451),IL15 (includes EG:16168),IFNB1 (includes EG:15977),JAK2,STAT1,IFNA1/IFNA13
IL-6 Signaling	1.79E00	0.016218101	1.21E-01	12.1	IL6ST,IL18RAP,SOCS1,IL1A,MAPK1,MAPK3,PIK3R1,JAK2,FOS,IL1RN,IL1B,CD14,PIK3R2,MAP2K1,MCL1
Interferon Signaling	1.19E01	1.25893E-12	5E-01	50	IFNG (includes EG:15978),IFIT3,SOCS1,OAS1,JAK1 (includes EG:16451),IFNB1 (includes EG:15977),IRF9,MX1,IFNGR1,IFI35,JAK2,PSMB8,TAP1,IFNA1/IFNA13,IFIT1,IFITM1,STAT2,STAT1
JAK/Stat Signaling	2.64E00	0.002290868	1.71E-01	17.1	SOCS1,FOS,JAK1 (includes EG:16451),MAPK1,MAPK3,PIK3R1,SOCS2,STAT2,PIK3R2,JAK2,STAT1,MAP2K1
PPAR Signaling	1.81E00	0.015488166	1.23E-01	12.3	IL18RAP,SRA1,IL1A,MAPK1,MAPK3,NR1H3,FOS,IL1RN,IL1B,PTGS2,RXRA,MAP2K1,SCAND1
Role of Cytokines in Mediating Communication between Immune Cells	5.41E00	3.89045E-06	2.73E-01	27.3	IFNG (includes EG:15978),IL1A,IFNA8,IL32,IL15 (includes EG:16168),IFNB1 (includes EG:15977),IL27,IFNA16,IFNA1/IFNA13,IL24,IFNA14,IL1RN,IFNA2,IL1B,IL23A
Role of Pattern Recognition Receptors in Recognition of Bacteria and Viruses	6.2E00	6.30957E-07	2.08E-01	20.8	OAS1,NLRP3,MAPK1,OAS2,MYD88,PIK3R1,MAPK3,IFNB1 (includes EG:15977),OAS3,IFNA1/IFNA13,IFIH1,IRF7,DDX58,CASP1,TLR7,NOD1,IL1B,PIK3R2,EIF2AK2,C3AR1,RIPK2,PRKCB
Role of IL-17A in Psoriasis	1.98E00	0.010471285	3.08E-01	30.8	CCL20,CXCL5,IL17RA,CXCL6
Role of IL-17F in Allergic Inflammatory Airway Diseases	3.19E00	0.000645654	2.17E-01	21.7	CXCL10,CCL7,MAPK1,MAPK3,IL1B,RPS6KA5,CXCL5,IL17RA,MAP2K1,CXCL6
Role of JAK family kinases in IL-6-type Cytokine Signaling	2.88E00	0.001318257	2.59E-01	25.9	IL6ST,SOCS1,JAK1 (includes EG:16451),MAPK1,MAPK3,JAK2,STAT1
Role of JAK1, JAK2 and TYK2 in Interferon Signaling	4.69E00	2.04174E-05	3.33E-01	33.3	SOCS1,IFNG (includes EG:15978),JAK1 (includes EG:16451),IFNB1 (includes EG:15977),IFNGR1,STAT2,JAK2,STAT1,IFNA1/IFNA13
Role of RIG1-like Receptors in Antiviral Innate Immunity	4.44E00	3.63078E-05	2.45E-01	24.5	DHX58,IFIH1,IRF7,RIPK1,IFNA8,DDX58,IFNB1 (includes EG:15977),IFNA2,IFNA16,IFNA1/IFNA13,IFNA14,TRIM25

TREM1 Signaling	5.69E00	2.04174E-06	2.25E-01	22.5	SIGIRR,ITGB1,TREM1,MAPK1,MYD88,MAPK3,LAT2,PLCG1,JAK2,FCGR2B,CD40,CCL7,CASP1,TLR7,CD86,IL1B

Adult poly I:C 6 h vs Lyovec

Ingenuity Canonical Pathways	-log(p-value)	p-value	Pathway enrichment score	Pathway enrichment score %	Molecules
Activation of IRF by Cytosolic Pattern Recognition Receptors	7.11E-01	0.194536008	1.39E-02	1.39	IFIT2
Crosstalk between Dendritic Cells and Natural Killer Cells	5.79E-01	0.263633139	1.05E-02	1.05	ACTG1
IL-6 Signaling	4.83E-01	0.328851631	8.06E-03	0.806	MCL1

Adult poly I:C 24 h vs Lyovec

Ingenuity Canonical Pathways	-log(p-value)	p-value	Pathway enrichment score	Pathway enrichment score %	Molecules
Activation of IRF by Cytosolic Pattern Recognition Receptors	9.24E00	5.7544E-10	1.94E-01	19.4	DHX58,ZBP1,IFNB1 (includes EG:15977),IRF9,ADAR,ISG15,IFIH1,IRF7,RIPK1,DDX58,IFNA2,STAT2,STAT1,IFIT2
Communication between Innate and Adaptive Immune Cells	1.49E00	0.032359366	5.45E-02	5.45	CXCL10,IL1A,IL1RN,TLR7,IFNB1 (includes EG:15977),TNFSF13B
Crosstalk between Dendritic Cells and Natural Killer Cells	2.08E00	0.008317638	7.37E-02	7.37	PRF1,MICB,ACTA2,TLR7,IFNB1 (includes EG:15977),TNFSF10,ACTG1
IL-10 Signaling	2.76E-01	0.529663444	2.56E-02	2.56	IL1A,IL1RN
IL-15 Production	1.5E00	0.031622777	9.68E-02	9.68	IFNB1 (includes EG:15977),JAK2,STAT1
IL-6 Signaling	4.5E-01	0.354813389	3.23E-02	3.23	SOCS1,IL1A,IL1RN,JAK2
Interferon Signaling	1.34E01	3.98107E-14	3.89E-01	38.9	IFIT3,SOCS1,OAS1,IFNB1 (includes EG:15977),IRF9,MX1,IFI35,PSMB8,JAK2,TAP1,IFIT1,IFITM1,STAT2,STAT1
JAK/Stat Signaling	1.02E00	0.095499259	5.71E-02	5.71	SOCS1,STAT2,JAK2,STAT1
PPAR Signaling	3.48E-01	0.44874539	2.83E-02	2.83	IL1A,IL1RN,PTGS2
Role of Cytokines in Mediating Communication between Immune Cells	1.86E00	0.013803843	9.09E-02	9.09	IL1A,IL1RN,IFNB1 (includes EG:15977),IFNA2,IL24
Role of IL-17A in Psoriasis	1.36E00	0.043651583	1.54E-01	15.4	CCL20,CXCL6
Role of IL-17F in Allergic Inflammatory Airway Diseases	5.13E-01	0.306902199	4.35E-02	4.35	CXCL10,CXCL6
Role of JAK family kinases in IL-6-type Cytokine Signaling	1.58E00	0.02630268	1.11E-01	11.1	SOCS1,JAK2,STAT1
Role of JAK1, JAK2 and TYK2 in Interferon Signaling	3.49E00	0.000323594	1.85E-01	18.5	SOCS1,IFNB1 (includes EG:15977),STAT2,JAK2,STAT1
Role of Pattern Recognition Receptors in Recognition of Bacteria and Viruses	4.42E00	3.80189E-05	1.04E-01	10.4	IFIH1,OAS1,IRF7,OAS2,MYD88,DDX58,TLR7,IFNB1 (includes EG:15977),NOD1,EIF2AK2,OAS3
Role of RIG1-like Receptors in Antiviral Innate Immunity	4.83E00	1.47911E-05	1.63E-01	16.3	DHX58,IFIH1,IRF7,RIPK1,DDX58,IFNB1 (includes EG:15977),IFNA2,TRIM25
TREM1 Signaling	1.18E00	0.066069345	5.63E-02	5.63	MYD88,TLR7,PLCG1,JAK2

Old LPS 6 h vs no treatment

Ingenuity Canonical Pathways	-log(p-value)	p-value	Pathway enrichment score	Pathway enrichment score %	Molecules
Activation of IRF by Cytosolic Pattern Recognition Receptors	8.4E00	3.98107E-09	3.06E-01	30.6	DHX58,MAP2K4,IL10,NFKBIE,ZBP1,IRF9,IL6,NFKB2,NFKB1,ADAR,ISG15,FADD,IFIH1,IRF7,RIPK1,NFKBIA,LTA,DDX58,STAT2,STAT1,IFIT2,TNF
Communication between Innate and Adaptive Immune Cells	3.66E00	0.000218776	1.73E-01	17.3	TLR1,IFNG (includes EG:15978),IL8,IL1A,IL10,IL15 (includes EG:16168),CD83,IL6,CCL3,HLA-F,CXCL10,IL18 (includes EG:16173),IL36G,IL1RN,CCL3L1/CCL3L3,IL1B,CSF2,TNF,CCR7
Crosstalk between Dendritic Cells and Natural Killer Cells	7.27E00	5.37032E-08	2.63E-01	26.3	KIR3DL1,TNFSF10,KIR3DL2,CD83,IL6,NFKB1,FAS,HLA-F,KIR3DL3/LOC100133046,PRF1,LTBR,IFNG (includes EG:15978),KIR2DL3 (includes others),IL3RA,TYROBP,CD69,IL15 (includes EG:16168),NFKB2,KIR2DL4/LOC100287534,IL18 (includes EG:16173),LTA,CSF2,TNF,CCR7,PVRL2
IL-10 Signaling	6.3E00	5.01187E-07	2.56E-01	25.6	CCR1,MAP2K4,IL18RAP,SOCS3,IL4R,IL1A,IL10,NFKBIE,IL6,NFKB2,NFKB1,IL1R2,HMOX1,IL18 (includes EG:16173),IL36G,NFKBIA,IL1RN,CD14,IL1B,TNF
IL-15 Production	2.22E00	0.006025596	2.26E-01	22.6	MAP3K11,IL15 (includes EG:16168),NFKB2,IL6,STAT1,NFKB1,IRF1 (includes EG:16362)
IL-6 Signaling	5.46E00	3.46737E-06	2.1E-01	21	MAP2K4,SOCS3,IL18RAP,SOCS1,IL1A,NFKBIE,MAPK3,IL6,NFKB1,IL1R2,IL36G,NFKBIA,NGFR,CSNK2A1,ABCB1,IL8,PIK3C2B,TNFRSF1A,NFKB2,CSNK2A2,IL18 (includes EG:16173),IL1RN,CD14,IL1B,TNFAIP6,TNF
Interferon Signaling	7.51E00	3.0903E-08	4.17E-01	41.7	IFNG (includes EG:15978),IFIT3,SOCS1,OAS1,PIAS1,IRF9,MX1,IFI35,PSMB8,TAP1,IRF1 (includes EG:16362),IFIT1,IFITM1,STAT2,STAT1
JAK/Stat Signaling	2.43E00	0.003715352	1.86E-01	18.6	SOCS1,SOCS3,PIK3C2B,STAT5A,PTPN6,MAPK3,PIAS1,IL6,STAT4,BCL2L1,PTPN1,STAT2,STAT1
PPAR Signaling	5.5E00	3.16228E-06	2.17E-01	21.7	PPARG,STAT5A,IL18RAP,IL1A,SRA1,TNFRSF1A,MED1 (includes EG:19014),NFKBIE,MAPK3,NFKB2,NFKB1,PDGFB,IL1R2,IL36G,IL18 (includes EG:16173),NFKBIA,IL1RN,NGFR,INS,IL1B,PTGS2,RXRA,TNF
Role of Cytokines in Mediating Communication between Immune Cells	3.83E00	0.000147911	2.55E-01	25.5	IL8,IFNG (includes EG:15978),IL1A,IL10,IL15 (includes EG:16168),IL6,CSF3,IL36G,IL18 (includes EG:16173),IL1RN,IL1B,IL23A,CSF2,TNF
Role of IL-17A in Psoriasis	1.7E00	0.019952623	3.08E-01	30.8	IL8,CXCL1,CCL20,CXCL6
Role of IL-17F in Allergic Inflammatory Airway Diseases	3.77E00	0.000169824	2.61E-01	26.1	CXCL10,IL8,MAPK3,CREB1,CXCL1,IL1B,NFKB2,IL6,RPS6KA1 (includes EG:20111),CSF2,NFKB1,CXCL6
Role of JAK family kinases in IL-6-type Cytokine Signaling	2.42E00	0.003801894	2.59E-01	25.9	MAP2K4,SOCS1,SOCS3,STAT5A,MAPK3,IL6,STAT1
Role of JAK1, JAK2 and TYK2 in Interferon Signaling	2.53E00	0.002951209	2.59E-01	25.9	SOCS1,IFNG (includes EG:15978),PTPN6,STAT2,NFKB2,STAT1,NFKB1
Role of Pattern Recognition Receptors in Recognition of Bacteria and Viruses	5.98E00	1.04713E-06	2.26E-01	22.6	MAP2K4,TLR1,PIK3C2B,OAS1,NLRP3,OAS2,IL10,MAPK3,IL6,NFKB2,NFKB1,OAS3,IFIH1,NOD2,IRF7,C5AR1,TICAM1,SYK,CREB1,DDX58,IL1B,EIF2AK2,TNF,RIPK2

Role of RIG1-like Receptors in Antiviral Innate Immunity	3.06E00	0.000870964	2.24E-01	22.4	DHX58,IFIH1,FADD,IRF7,RIPK1,NFKBIA,NFKBIE,DDX58,NFKB2,NFKB1,TRIM25
TREM1 Signaling	6.7E00	1.99526E-07	2.68E-01	26.8	TLR1,STAT5A,IL8,TREM1,TYROBP,IL10,MAPK3,LAT2,MPO,CD83,IL6,NFKB2,CCL3,NFKB1,IL18 (includes EG:16173),NOD2,IL1B,CSF2,TNF

Old LPS 24 h vs no treatment

Ingenuity Canonical Pathways	-log(p-value)	p-value	Pathway enrichment score	Pathway enrichment score %	Molecules
Activation of IRF by Cytosolic Pattern Recognition Receptors	7.77E-01	0.167109061	1.39E-01	13.9	MAP2K4,FADD,RELA,IRF7,IL10,NFKBIE,IRF9,NFKB2,IL6,ADAR
Communication between Innate and Adaptive Immune Cells	2.56E00	0.002754229	1.82E-01	18.2	TLR1,IL8,IL1A,IL10,CD4,TLR8,HLA-DRB3 (includes others),CD83,IL6,IGHG1,CCL3,HLA-F,CXCL10,IL36G,IL18 (includes EG:16173),CCL3L1/CCL3L3,IL1B,CSF2,CCR7,TNFSF13B
Crosstalk between Dendritic Cells and Natural Killer Cells	3.56E00	0.000275423	2.32E-01	23.2	RELA,KIR3DL1,KIR2DL3 (includes others),TYROBP,KIR3DL2,HLA-DRB3 (includes others),CD83,IL6,NFKB2,IL2RB,KIR2DL4/LOC100287534,HLA-F,KIR3DL3/LOC100133046,IL18 (includes EG:16173),KIR2DL5A,MICB,FSCN1,LTBR,CSF2,CCR7,PVRL2,CAMK2B
IL-10 Signaling	6.79E00	1.62181E-07	3.08E-01	30.8	CCR1,MAP2K4,RELA,IL18RAP,SOCS3,CCR5,IL1A,MAPK1,BLVRA,IL10,FCGR2A,NFKBIE,BLVRB,NFKB2,IL6,FCGR2B,IL1R2,FOS,IL36G,IL18 (includes EG:16173),SP1,MAP3K7 (includes EG:172842),CD14,IL1B
IL-15 Production	1.57E00	0.026915348	2.26E-01	22.6	RELA,MAP3K11,PTK2B (includes EG:19229),NFKB2,IL6,PRKCZ,IRF1 (includes EG:16362)
IL-6 Signaling	4.59E00	2.5704E-05	2.34E-01	23.4	MAP2K4,RELA,SOCS3,RAF1,IL18RAP,SOCS1,IL1A,MAPK1,MAPK3,NFKBIE,HRAS,IL6,IL1R2,IL36G,MAP3K7 (includes EG:172842),CSNK2A1,MCL1,IL8,PIK3C2B,RRAS,GRB2,TNFRSF1A,NFKB2,CSNK2A2,FOS,IL18 (includes EG:16173),CD14,IL1B,HSPB1
Interferon Signaling	1.49E00	0.032359366	2.22E-01	22.2	SOCS1,RELA,PIAS1,IFITM1,IFNGR1,IRF9,IFNAR2,IRF1 (includes EG:16362)
JAK/Stat Signaling	2.17E00	0.00676083	2.14E-01	21.4	SOCS1,RAF1,SOCS3,PIK3C2B,PTPN6,MAPK1,GRB2,RRAS,MAPK3,PIAS1,HRAS,IL6,FOS,BCL2L1,MTOR
PPAR Signaling	5.43E00	3.71535E-06	2.55E-01	25.5	RELA,IL18RAP,RAF1,IL1A,MAPK1,NFKBIE,MAPK3,HRAS,PDGFC,IL1R2,IL36G,MAP3K7 (includes EG:172842),CITED2,SRA1,MED1 (includes EG:19014),RRAS,PPARD,GRB2,TNFRSF1A,NR1H3,NFKB2,PDGFB,FOS,IL18 (includes EG:16173),IL1B,PTGS2,RXRA
Role of Cytokines in Mediating Communication between Immune Cells	1.39E00	0.040738028	2E-01	20	IL8,IL36G,IL1A,IL18 (includes EG:16173),IL10,IL1B,IL6,IL23A,CSF2,IL24,IL25
Role of IL-17A in Psoriasis	2.76E00	0.001737801	4.62E-01	46.2	IL8,CXCL1,CCL20,S100A8,IL17RA,CXCL6
Role of IL-17F in Allergic Inflammatory Airway Diseases	6.52E00	3.01995E-07	3.91E-01	39.1	RELA,IL8,RAF1,MAPK1,MAPK3,CXCL1,IL6,NFKB2,IL17RA,CXCL6,CXCL10,CCL2,CCL7,CREB1,IL1B,RPS6KA5,RPS6KA2,CSF2
Role of JAK family kinases in IL-6-type Cytokine Signaling	1.26E00	0.054954087	2.22E-01	22.2	MAP2K4,SOCS1,SOCS3,MAPK1,MAPK3,IL6
Role of JAK1, JAK2 and TYK2 in Interferon Signaling	1.85E00	0.014125375	2.59E-01	25.9	SOCS1,RAF1,RELA,PTPN6,IFNGR1,NFKB2,IFNAR2
Role of Pattern Recognition Receptors in Recognition of Bacteria and Viruses	3.07E00	0.000851138	2.08E-01	20.8	MAP2K4,TLR1,RELA,PIK3C2B,NLRP3,PRKCQ,MAPK1,IL10,MYD88,MAPK3,TLR8,IL6,NFKB2,PRKCZ,IRF7,C5AR1,SYK,CREB1,IL1B,C3AR1,RIPK2,PRKCB
Role of RIG1-like Receptors in Antiviral Innate Immunity	2.5E-01	0.562341325	1.02E-01	10.2	FADD,RELA,IRF7,NFKBIE,NFKB2
TREM1 Signaling	8.19E00	6.45654E-09	3.38E-01	33.8	TLR1,RELA,IL8,MAPK1,TYROBP,GRB2,IL10,MYD88,MAPK3,LAT2,TLR8,PLCG1,ITGA5,CD83,NFKB2,IL6,CCL3,FCGR2B,IL18 (includes EG:16173),CCL2,CCL7,IL1B,CSF2,ITGAX

Old CLO97 6 h vs no treatment

Ingenuity Canonical Pathways	-log(p-value)	p-value	Pathway enrichment score	Pathway enrichment score %	Molecules
Activation of IRF by Cytosolic Pattern Recognition Receptors	8.89E00	1.28825E-09	2.78E-01	27.8	DHX58,ZBP1,IRF9,IL6,NFKB2,NFKB1,ADAR,ISG15,IFIH1,IRF7,NFKBIA,RIPK1,CD40,LTA,DDX58,STAT2,CHUK,STAT1,IFIT2,TNF
Communication between Innate and Adaptive Immune Cells	5.88E00	1.31826E-06	1.82E-01	18.2	IFNG (includes EG:15978),IL8,IL1A,IL15 (includes EG:16168),CD83,IL6,CCL3,HLA-F,CXCL10,IL36G,CCL4,CD40,IL1RN,HLA-E,CCL3L1/CCL3L3,IL1B,CSF2,TNF,TNFSF13B,CCR7
Crosstalk between Dendritic Cells and Natural Killer Cells	9.66E00	2.18776E-10	2.63E-01	26.3	IL15RA,ACTA2,TNFSF10,KIR3DL2,CD83,IL6,NFKB1,IL2RB,FAS,HLA-F,PRF1,MICB,TNFRSF1B,FASLG,IFNG (includes EG:15978),CD69,IL15 (includes EG:16168),NFKB2,KIR2DL4/LOC100287534,CD40,HLA-E,LTA,CSF2,TNF,CCR7
IL-10 Signaling	4.02E00	9.54993E-05	1.79E-01	17.9	IL18RAP,SOCS3,IL1A,NFKB2,IL6,NFKB1,HMOX1,IL36G,NFKBIA,IL1RN,CD14,IL1B,CHUK,TNF
IL-15 Production	2.18E00	0.006606934	1.94E-01	19.4	IL15 (includes EG:16168),NFKB2,IL6,STAT1,NFKB1,IRF1 (includes EG:16362)
IL-6 Signaling	4.29E00	5.12861E-05	1.61E-01	16.1	IL6ST,IL8,IL18RAP,SOCS1,SOCS3,IL1A,IL6,NFKB2,NFKB1,SHC1 (includes EG:20416),IL36G,NFKBIA,IL1RN,NGFR,CD14,IL1B,CHUK,TNFAIP6,TNFRSF1B,TNF
Interferon Signaling	9.11E00	7.76247E-10	4.17E-01	41.7	IFNG (includes EG:15978),IFIT3,SOCS1,OAS1,PIAS1,IRF9,MX1,IFI35,PSMB8,TAP1,IRF1 (includes EG:16362),IFIT1,IFITM1,STAT2,STAT1
JAK/Stat Signaling	2.44E00	0.003630781	1.57E-01	15.7	STAT4,SHC1 (includes EG:20416),BCL2L1,SOCS1,SOCS3,STAT5A,PIAS1,PTPN1,STAT2,IL6,STAT1
PPAR Signaling	7.59E00	2.5704E-08	2.17E-01	21.7	PPARG,STAT5A,IL18RAP,IL1A,SRA1,NFKB2,NFKB1,PDGFB,SHC1 (includes EG:20416),IL36G,NFKBIA,HSP90AB1,IL1RN,NGFR,INS,HSP90AA1,IL1B,CHUK,NCOR2,PTGS2,TNFRSF1B,RXRA,TNF
Role of Cytokines in Mediating Communication between Immune Cells	2.6E00	0.002511886	1.82E-01	18.2	IL8,IFNG (includes EG:15978),IL36G,IL1A,IL1RN,IL15 (includes EG:16168),IL1B,IL6,CSF2,TNF
Role of IL-17A in Psoriasis	1.32E00	0.047863009	2.31E-01	23.1	IL8,CCL20,CXCL6
Role of IL-17F in Allergic Inflammatory Airway Diseases	4.92E00	1.20226E-05	2.61E-01	26.1	CXCL10,IL8,CCL4,CCL2,CCL7,IL1B,NFKB2,IL6,RPS6KA1 (includes EG:20111),CSF2,NFKB1,CXCL6
Role of JAK family kinases in IL-6-type Cytokine Signaling	2.35E00	0.004466836	2.22E-01	22.2	IL6ST,SOCS1,SOCS3,STAT5A,IL6,STAT1
Role of JAK1, JAK2 and TYK2 in Interferon Signaling	2.45E00	0.003548134	2.22E-01	22.2	SOCS1,IFNG (includes EG:15978),STAT2,NFKB2,STAT1,NFKB1
Role of Pattern Recognition Receptors in Recognition of Bacteria and Viruses	3.89E00	0.000128825	1.6E-01	16	OAS1,NLRP3,OAS2,MYD88,IL6,NFKB2,OAS3,NFKB1,IFIH1,IRF7,C5AR1,TICAM1,DDX58,IL1B,EIF2AK2,TNF,RIPK2
Role of RIG1-like Receptors in Antiviral Innate Immunity	3.39E00	0.00040738	2.04E-01	20.4	DHX58,IFIH1,IRF7,RIPK1,NFKBIA,DDX58,CHUK,NFKB2,NFKB1,TRIM25
TREM1 Signaling	5.45E00	3.54813E-06	2.11E-01	21.1	IL8,STAT5A,MYD88,MPO,CD83,IL6,NFKB2,CCL3,NFKB1,CCL2,CCL7,CD40,IL1B,CSF2,TNF

Old CLO97 24 h vs no treatment

Ingenuity Canonical Pathways	-log(p-value)	p-value	Pathway enrichment score	Pathway enrichment score %	Molecules
Activation of IRF by Cytosolic Pattern Recognition Receptors	6.4E00	3.98107E-07	2.92E-01	29.2	DHX58,IL10,ZBP1,TBK1,IRF9,IL6,NFKB2,NFKB1,ADAR,ISG15,TANK,IFIH1,IRF7,NFKBIA,CD40,LTA,DDX58,STAT2,STAT1,IFIT2,TNF
Communication between Innate and Adaptive Immune Cells	4.56E00	2.75423E-05	2.09E-01	20.9	IFNG (includes EG:15978),IL8,IL1A,IL10,IL15 (includes EG:16168),CD83,IL6,CCL3,HLA-F,TLR10,IL36G,CD40,CD80 (includes EG:12519),HLA-E,IL1RN,IL36RN,CCL3L1/CCL3L3,IL1B,CSF2,TNFRSF13B,TNF,CCR7,IL4 (includes EG:16189)
Crosstalk between Dendritic Cells and Natural Killer Cells	9.69E00	2.04174E-10	3.26E-01	32.6	KIR3DL1,IL15RA,KIR3DL2,TNFSF10,CD83,IL6,NFKB1,IL2RB,HLA-F,KIR2DL5A,CD226,LTBR,TNFRSF1B,IL4 (includes EG:16189),KIR2DL3 (includes others),IFNG (includes EG:15978),IL2RG,IL3RA,ACTB,IL15 (includes EG:16168),NFKB2,KIR2DL4/LOC100287534,ACTG1,CD40,CD80 (includes EG:12519),HLA-E,FSCN1,LTA,CSF2,TNF,CCR7
IL-10 Signaling	4.6E00	2.51189E-05	2.44E-01	24.4	SOCS3,CCR5,IL1A,IL10,FCGR2A,BLVRB,IL6,STAT3,NFKB2,NFKB1,IL1R2,FOS,IL36G,NFKBIA,IL1RN,IL36RN,IL1B,ELK1,TNF
IL-15 Production	1.33E00	0.046773514	1.94E-01	19.4	IL15 (includes EG:16168),NFKB2,IL6,STAT1,NFKB1,IRF1 (includes EG:16362)
IL-6 Signaling	2.62E00	0.002398833	1.77E-01	17.7	IL8,SOCS1,SOCS3,IL1A,IL6,NFKB2,STAT3,NFKB1,IL1R2,FOS,TRAF2,IL36G,NFKBIA,IL1RN,IL36RN,IL1B,PIK3CD,TNFAIP6,TNFRSF1B,ELK1,TNF,MCL1
Interferon Signaling	8.38E00	4.16869E-09	4.72E-01	47.2	IFNG (includes EG:15978),IFIT3,SOCS1,OAS1,PTPN2,IRF9,MX1,IFNGR1,IFI35,PSMB8,TAP1,IRF1 (includes EG:16362),BCL2,IFIT1,IFITM1,STAT2,STAT1
JAK/Stat Signaling	1.84E00	0.014454398	1.86E-01	18.6	SOCS1,SOCS3,STAT5A,PTPN6,PIAS2,STAT3,IL6,FOS,CDKN1A,PTPN1,STAT2,PIK3CD,STAT1
PPAR Signaling	3.86E00	0.000138038	2.08E-01	20.8	STAT5A,SRA1,IL1A,MED1 (includes EG:19014),NFKB2,NFKB1,PDGFB,IL1R2,FOS,TRAF2,IL36G,NFKBIA,HSP90AB1,IL1RN,IL36RN,HSP90AA1,IL1B,NCOR2,PTGS2,TNFRSF1B,PDGFD,TNF
Role of Cytokines in Mediating Communication between Immune Cells	3.09E00	0.000812831	2.55E-01	25.5	IL8,IFNG (includes EG:15978),IL1A,IL10,IL15 (includes EG:16168),IL6,IL24,IL36G,IL1RN,IL36RN,IL1B,CSF2,TNF,IL4 (includes EG:16189)
Role of IL-17A in Psoriasis	1.47E00	0.033884416	3.08E-01	30.8	IL8,CXCL1,CCL20,CXCL6
Role of IL-17F in Allergic Inflammatory Airway Diseases	3.11E00	0.000776247	2.61E-01	26.1	IL8,TRAF3IP2,CCL2,CCL7,CXCL1,ATF4,IL1B,NFKB2,IL6,CSF2,NFKB1,CXCL6
Role of JAK family kinases in IL-6-type Cytokine Signaling	1.48E00	0.033113112	2.22E-01	22.2	SOCS1,SOCS3,STAT5A,STAT3,IL6,STAT1
Role of JAK1, JAK2 and TYK2 in Interferon Signaling	4.29E00	5.12861E-05	3.7E-01	37	SOCS1,IFNG (includes EG:15978),PTPN6,PTPN2,IFNGR1,STAT2,STAT3,NFKB2,STAT1,NFKB1

Role of Pattern Recognition Receptors in Recognition of Bacteria and Viruses	2.55E00	0.002818383	1.79E-01	17.9	OAS1,OAS2,IL10,IL6,NFKB2,C1QB,NFKB1,OAS3,IFIH1,IRF7,TICAM1,SYK,DDX58,CASP1,IL1B,PIK3CD,EIF2AK2,TNF,RIPK2
Role of RIG1-like Receptors in Antiviral Innate Immunity	2.48E00	0.003311311	2.24E-01	22.4	DHX58,TANK,IFIH1,TRAF2,IRF7,NFKBIA,DDX58,TBK1,NFKB2,NFKB1,TRIM25
TREM1 Signaling	4.96E00	1.09648E-05	2.54E-01	25.4	SIGIRR,STAT5A,IL8,IL10,CD83,IL6,STAT3,NFKB2,NFKB1,CCL3,TLR10,CCL7,CD40,CCL2,CASP1,IL1B,CSF2,TNF

Old 5'pppRNA 6 h vs Lyovec

Ingenuity Canonical Pathways	-log(p-value)	p-value	Pathway enrichment score	Pathway enrichment score %	Molecules
Activation of IRF by Cytosolic Pattern Recognition Receptors	1.33E00	0.046773514	1.25E-01	12.5	MAP2K4,IFIH1,IFNA8,DDX58,CREBBP,IFNB1 (includes EG:15977),PIN1,IFIT2,ISG15
Communication between Innate and Adaptive Immune Cells	2.58E-01	0.552077439	6.36E-02	6.36	CXCL10,CD80 (includes EG:12519),IFNB1 (includes EG:15977),CD86,IGHG1,CD8A,CCL15
IL-10 Signaling	5.93E-01	0.25527013	8.97E-02	8.97	MAP2K4,FOS,IL4R,JAK1 (includes EG:16451),MAPK14,MAP2K3,IL1RAP
IL-6 Signaling	2.83E-01	0.521194711	7.26E-02	7.26	MAP2K4,FOS,MAPK14,PIK3R1,SRF,MAP2K3,MAP2K1,IL1RAP, ATM
Interferon Signaling	1.91E00	0.012302688	1.94E-01	19.4	IFIT3,IFIT1,OAS1,JAK1 (includes EG:16451),IFNB1 (includes EG:15977),IFNGR1,IFI35
JAK/Stat Signaling	6.13E-01	0.243781082	1E-01	10	FOS,JAK1 (includes EG:16451),PIK3R1,SOCS4,SOCS7,MAP2K1,ATM
Role of IL-17F in Allergic Inflammatory Airway Diseases	3.84E-01	0.413047502	8.7E-02	8.7	CXCL10,ATF4,CREB5,MAP2K1
Role of JAK family kinases in IL-6-type Cytokine Signaling	5.32E-01	0.293764965	1.11E-01	11.1	MAP2K4,JAK1 (includes EG:16451),MAPK14
Role of JAK1, JAK2 and TYK2 in Interferon Signaling	5.65E-01	0.272270131	1.11E-01	11.1	JAK1 (includes EG:16451),IFNB1 (includes EG:15977),IFNGR1
Role of Pattern Recognition Receptors in Recognition of Bacteria and Viruses	9.58E-01	0.110153931	1.04E-01	10.4	MAP2K4,IFIH1,OAS1,OAS2,PIK3R1,DDX58,IFNB1 (includes EG:15977),PRKCH,EIF2AK2,OAS3,ATM
Role of RIG1-like Receptors in Antiviral Innate Immunity	6.22E-01	0.238781128	1.02E-01	10.2	IFIH1,IFNA8,DDX58,CREBBP,IFNB1 (includes EG:15977)

Old 5'pppRNA 24 h vs Lyovec

Ingenuity Canonical Pathways	-log(p-value)	p-value	Pathway enrichment score	Pathway enrichment score %	Molecules
Activation of IRF by Cytosolic Pattern Recognition Receptors	1.19E01	1.25893E-12	3.33E-01	33.3	DHX58,IL10,ZBP1,IFNB1 (includes EG:15977),IRF9,IKBKE,ADAR,IFNA10,IFNA16,IFNA14,IFNA1/IFNA13,ISG15,TANK,IFIH1,IRF7,RIPK1,CD40,LTA,IFNA7,DDX58,IFNA2,STAT2,STAT1,IFIT2
Communication between Innate and Adaptive Immune Cells	5.45E00	3.54813E-06	1.82E-01	18.2	IFNG (includes EG:15978),IL8,IL1A,IL10,IL15 (includes EG:16168),IFNB1 (includes EG:15977),IFNA1/IFNA13,HLA-F,CXCL10,CD40,HLA-A,HLA-E,IL1RN,TLR7,CCL3L1/CCL3L3,IL1B,HLA-G,TNFRSF13B,TNFSF13B,CCR7
Crosstalk between Dendritic Cells and Natural Killer Cells	6.97E00	1.07152E-07	2.32E-01	23.2	IFNG (includes EG:15978),IL15RA,KLRD1,CD69,IL15 (includes EG:16168),ACTA2,IFNB1 (includes EG:15977),TNFSF10,IFNA1/IFNA13,FAS,HLA-F,PRF1,HLA-A,MICB,CD40,HLA-E,LTA,FSCN1,TLR7,HLA-G,CCR7,PVRL2
IL-10 Signaling	4.32E00	4.7863E-05	1.92E-01	19.2	CCR1,IL18RAP,IL1A,BLVRA,IL10,FCGR2A,IKBKE,FOS,SP1,IL1RN,IL10RB,IL10RA,CD14,IL1B,MAP2K3
IL-15 Production	3.47E00	0.000338844	2.58E-01	25.8	PRKCI,TWF1 (includes EG:19230),IL15 (includes EG:16168),IFNB1 (includes EG:15977),JAK2,STAT1,IFNA1/IFNA13,IRF1 (includes EG:16362)
IL-6 Signaling	2.22E00	0.006025596	1.29E-01	12.9	IL8,IL18RAP,SOCS1,PIK3C2B,IL1A,GRB2,IKBKE,JAK2,FOS,SHC1 (includes EG:20416),IL1RN,IL1B,CD14,MAP2K3,MCL1,HSPB1
Interferon Signaling	1.09E01	1.25893E-11	4.72E-01	47.2	IFNG (includes EG:15978),IFIT3,SOCS1,OAS1,IFNB1 (includes EG:15977),IRF9,MX1,IFI35,JAK2,PSMB8,IFNA1/IFNA13,TAP1,IRF1 (includes EG:16362),IFIT1,IFITM1,STAT2,STAT1
JAK/Stat Signaling	2.23E00	0.005888437	1.57E-01	15.7	SHC1 (includes EG:20416),SOCS1,PIK3C2B,FOS,STAT5A,PIAS4,GRB2,STAT2,JAK2,STAT1,SOCS5
PPAR Signaling	2.26E00	0.005495409	1.32E-01	13.2	IL18RAP,STAT5A,IL1A,GRB2,PDGFA,NR1H3,IKBKE,SHC1 (includes EG:20416),FOS,IL1RN,IL1B,PTGS2,RXRA,SCAND1
Role of Cytokines in Mediating Communication between Immune Cells	7.05E00	8.91251E-08	3.09E-01	30.9	IFNG (includes EG:15978),IL8,IL1A,IL10,IL15 (includes EG:16168),IFNB1 (includes EG:15977),IL27,IFNA10,IFNA16,IFNA1/IFNA13,IL24,IFNA14,IL1RN,IFNA7,IFNA2,IL1B,IL23A

Role of IL-17A in Psoriasis	2.9E00	0.001258925	3.85E-01	38.5	IL8,CCL20,S100A8,CXCL5,CXCL6
Role of IL-17F in Allergic Inflammatory Airway Diseases	1.58E00	0.02630268	1.52E-01	15.2	CXCL10,IL8,CCL7,IL1B,RPS6KA5,CXCL5,CXCL6
Role of JAK family kinases in IL-6-type Cytokine Signaling	1.05E00	0.089125094	1.48E-01	14.8	SOCS1,STAT5A,JAK2,STAT1
Role of JAK1, JAK2 and TYK2 in Interferon Signaling	3.05E00	0.000891251	2.59E-01	25.9	SOCS1,IFNG (includes EG:15978),IFNB1 (includes EG:15977),STAT2,JAK2,STAT1,IFNA1/IFNA13
Role of Pattern Recognition Receptors in Recognition of Bacteria and Viruses	5.15E00	7.07946E-06	1.89E-01	18.9	PIK3C2B,OAS1,OAS2,IL10,MYD88,IFNB1 (includes EG:15977),OAS3,IFNA1/IFNA13,IFIH1,IRF7,PRKCI,TICAM1,DDX58,CASP1,TLR7,NOD1,IL1B,EIF2AK2,C3AR1,RIPK2
Role of RIG1-like Receptors in Antiviral Innate Immunity	6.92E00	1.20226E-07	3.06E-01	30.6	DHX58,IFNB1 (includes EG:15977),IKBKE,IFNA10,IFNA16,IFNA1/IFNA13,IFNA14,TANK,IFIH1,IRF7,RIPK1,DDX58,IFNA7,IFNA2,TRIM25
TREM1 Signaling	3.81E00	0.000154882	1.83E-01	18.3	ITGB1,IL8,STAT5A,GRB2,IL10,MYD88,LAT2,JAK2,CCL7,CD40,TLR7,CASP1,IL1B

Old poly I:C 6 h vs Lyovec

Ingenuity Canonical Pathways	-log(p-value)	p-value	Pathway enrichment score	Pathway enrichment score %	Molecules
no pathways					

Old poly I:C 24 h vs Lyovec

Ingenuity Canonical Pathways	-log(p-value)	p-value	Pathway enrichment score	Pathway enrichment score %	Molecules
Activation of IRF by Cytosolic Pattern Recognition Receptors	5.39E00	4.0738E-06	2.08E-01	20.8	DHX58,CREBBP,ZBP1,IFNB1 (includes EG:15977),ADAR,ISG15,TANK,IFIH1,IRF7,RIPK1,LTA,DDX58,STAT2,STAT1,IFIT2
Communication between Innate and Adaptive Immune Cells	2.01E00	0.009772372	1.09E-01	10.9	CXCL10,IL8,IL1A,TLR7,IFNB1 (includes EG:15977),IL36A,CCL3L1/CCL3L3,IL1B,IL1F10,IL37,TNFSF13B,IL36B
Crosstalk between Dendritic Cells and Natural Killer Cells	1.72E00	0.019054607	1.16E-01	11.6	IL3RA,MICB,LTA,CD69,ACTB,ACTA2,TLR7,IFNB1 (includes EG:15977),TNFSF10,FAS,FASLG
IL-10 Signaling	3.13E00	0.00074131	1.54E-01	15.4	IL1R2,HMOX1,IL1A,BLVRA,IL1RL1,IL36A,IL1B,IL1F10,STAT3,IL37,TAB1,IL36B
IL-15 Production	6.48E-01	0.224905461	9.68E-02	9.68	IFNB1 (includes EG:15977),JAK2,STAT1
IL-6 Signaling	3.2E00	0.000630957	1.37E-01	13.7	IL8,SOCS1,IL1A,IL1RL1,IL36A,IL37,IL1F10,STAT3,JAK2,IL36B,IL1R2,CSNK2A1,MRAS,IL1B,PIK3CD,PIK3R2,TAB1
Interferon Signaling	6.42E00	3.80189E-07	3.33E-01	33.3	IFIT3,SOCS1,IFIT1,OAS1,IFNB1 (includes EG:15977),IFITM1,MX1,IFI35,STAT2,PSMB8,JAK2,STAT1
JAK/Stat Signaling	1.71E00	0.019498446	1.29E-01	12.9	SOCS1,MRAS,STAT2,PIK3CD,STAT3,PIK3R2,JAK2,STAT1,CCKBR
PPAR Signaling	1.9E00	0.012589254	1.13E-01	11.3	IL1R2,IL1A,IL1RL1,CREBBP,MRAS,IL36A,IL1B,IL1F10,PTGS2,IL37,TAB1,IL36B
Role of Cytokines in Mediating Communication between Immune Cells	2.25E00	0.005623413	1.64E-01	16.4	IL8,IL1A,IFNB1 (includes EG:15977),IL36A,IL1B,IL1F10,IL37,IL24,IL36B
Role of IL-17A in Psoriasis	2.2E00	0.006309573	3.08E-01	30.8	IL8,CCL20,CXCL5,CXCL6
Role of IL-17F in Allergic Inflammatory Airway Diseases	1.35E00	0.044668359	1.3E-01	13	CXCL10,IL8,IL1B,RPS6KA5,CXCL5,CXCL6
Role of JAK family kinases in IL-6-type Cytokine Signaling	1.21E00	0.0616595	1.48E-01	14.8	SOCS1,STAT3,JAK2,STAT1
Role of JAK1, JAK2 and TYK2 in Interferon Signaling	2.58E00	0.002630268	2.22E-01	22.2	SOCS1,IFNB1 (includes EG:15977),STAT2,STAT3,JAK2,STAT1
Role of RIG1-like Receptors in Antiviral Innate Immunity	2.95E00	0.001122018	1.84E-01	18.4	DHX58,TANK,IFIH1,IRF7,RIPK1,DDX58,CREBBP,IFNB1 (includes EG:15977),TRIM25
TREM1 Signaling	2.05E00	0.008912509	1.27E-01	12.7	SIGIRR,ITGB1,IL8,MYD88,TLR7,CASP1,IL1B,STAT3,JAK2
