# Supplementary material for: Global analyses revealed age-related alterations in innate immune responses after stimulation of pathogen recognition receptors
Source: Aging Cell. 2015 Feb 27;14(3):421–32. doi: 10.1111/acel.12320 (PMC4406671; doi:10.1111/acel.12320)
Supplement: Supplementary file 1 [file acel0014-0421-sd1.zip › SuppInform Table 7 Adult and Old Cohort.docx]

**Supplementary Table 7:** Description of the adult and old cohorts.

|  | **Adult (*n* = 31)** | **Old (*n* = 31)** |
| --- | --- | --- |
| **Ave Age (Range)** | 30 (21-39) | 74 (65-87) |
| **Gender M/F (female %)** | 18/13 (42%) | 16/15 (48%) |
| **Race** |  |  |
| White (Non-Hispanics) | 17 | 26 |
| White (Hispanics) | 11 | 1 |
| African-American | 1 | 2 |
| Other (Non-Hispanics) | 2 |  |
| Others (Hispanics) |  | 2 |
| **Comorbidities** | 4 (13%) | 24 (78%) |
| None | 27 (87%) | 7 (22%) |
| Hypothyroid | 1 (3%) |  |
| Diabetes mellitus | 1 (3%) | 2 (6%) |
| Arthritis | 2 (6%) | 14 (45%) |
| ET Neurological |  | 1 (3%) |
| Hypertension |  | 15 (48%) |
| Stroke |  | 4 (13%) |
| Heart Attack |  | 2 (6%) |
| Bronchitis |  | 3 (10%) |
| Vasculitis |  | 1 (3%) |
| **Medications** |  |  |
| Prescription | 3 (10%) | 20 (64%) |
| Over-the-counter | 9 (29%) | 18 (58%) |
