# Supplementary figures and images for: Global analyses revealed age-related alterations in innate immune responses after stimulation of pathogen recognition receptors
Source: Aging Cell. 2015 Feb 27;14(3):421–32. doi: 10.1111/acel.12320 (PMC4406671; doi:10.1111/acel.12320)

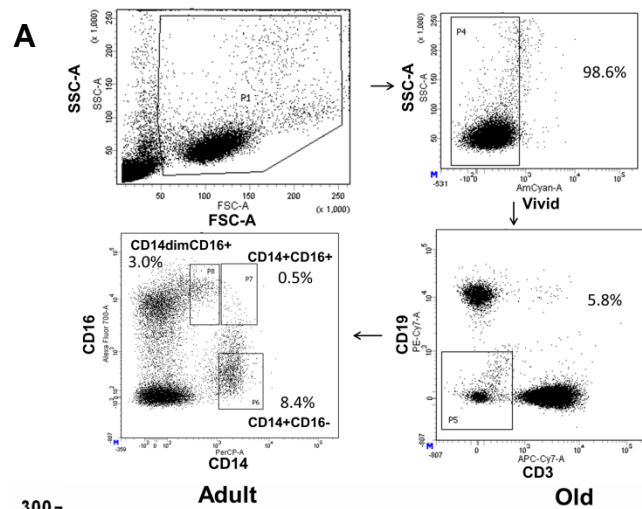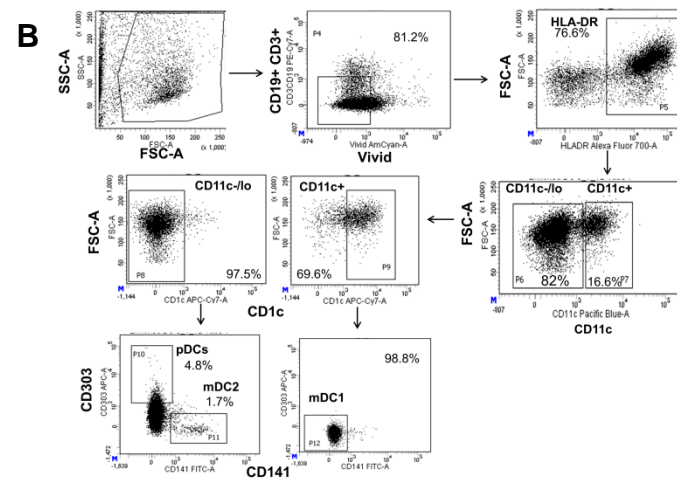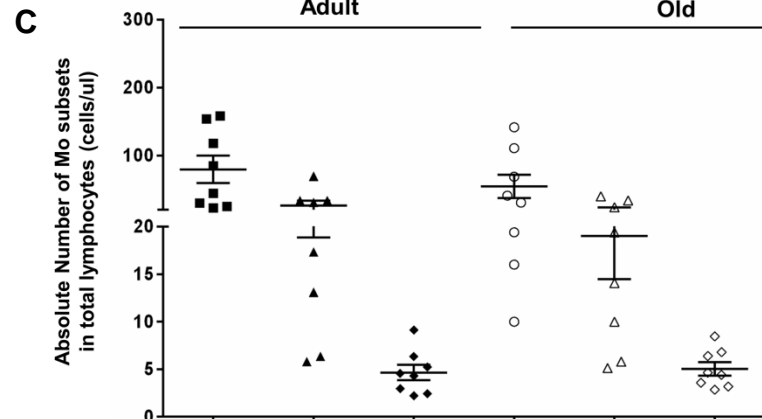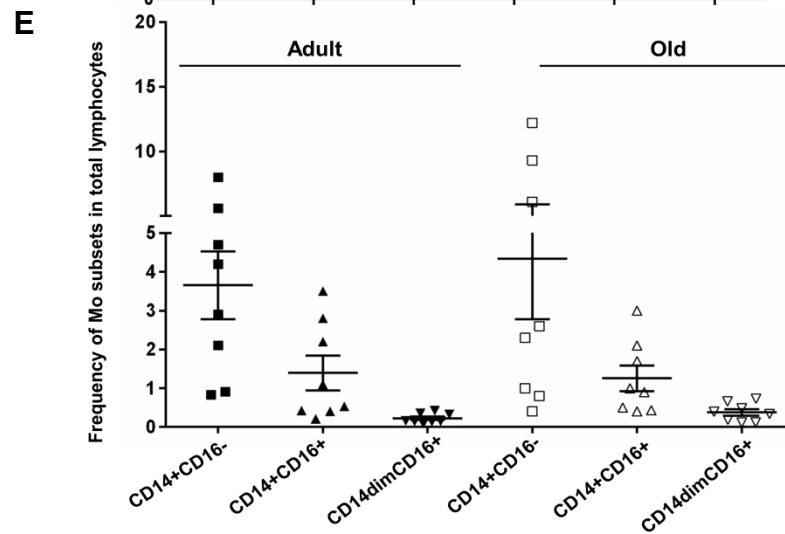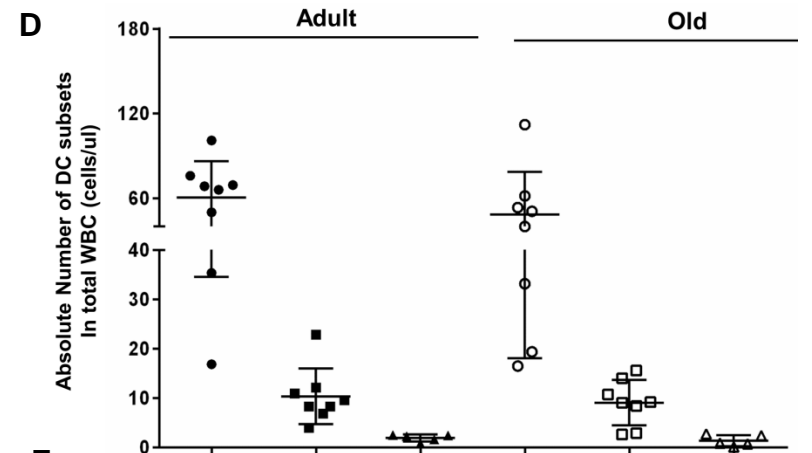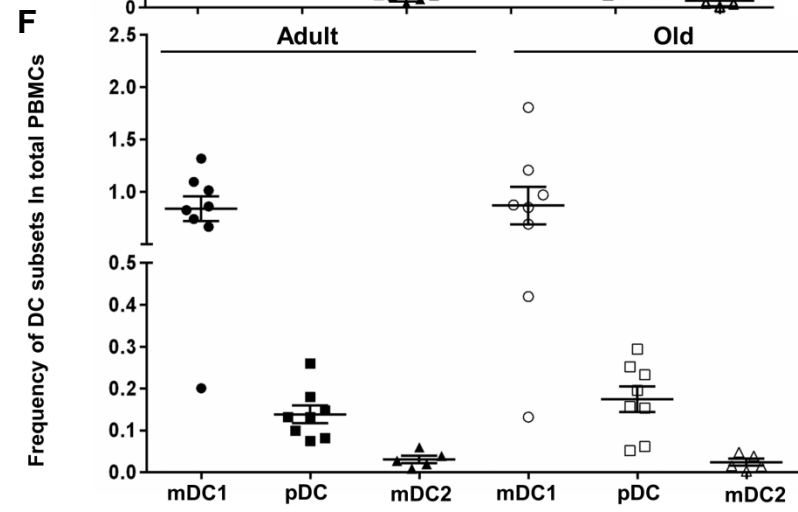

Supplement: Supplementary file 1 [file acel0014-0421-sd1.zip › SuppInform Figure 1 rev.pdf]

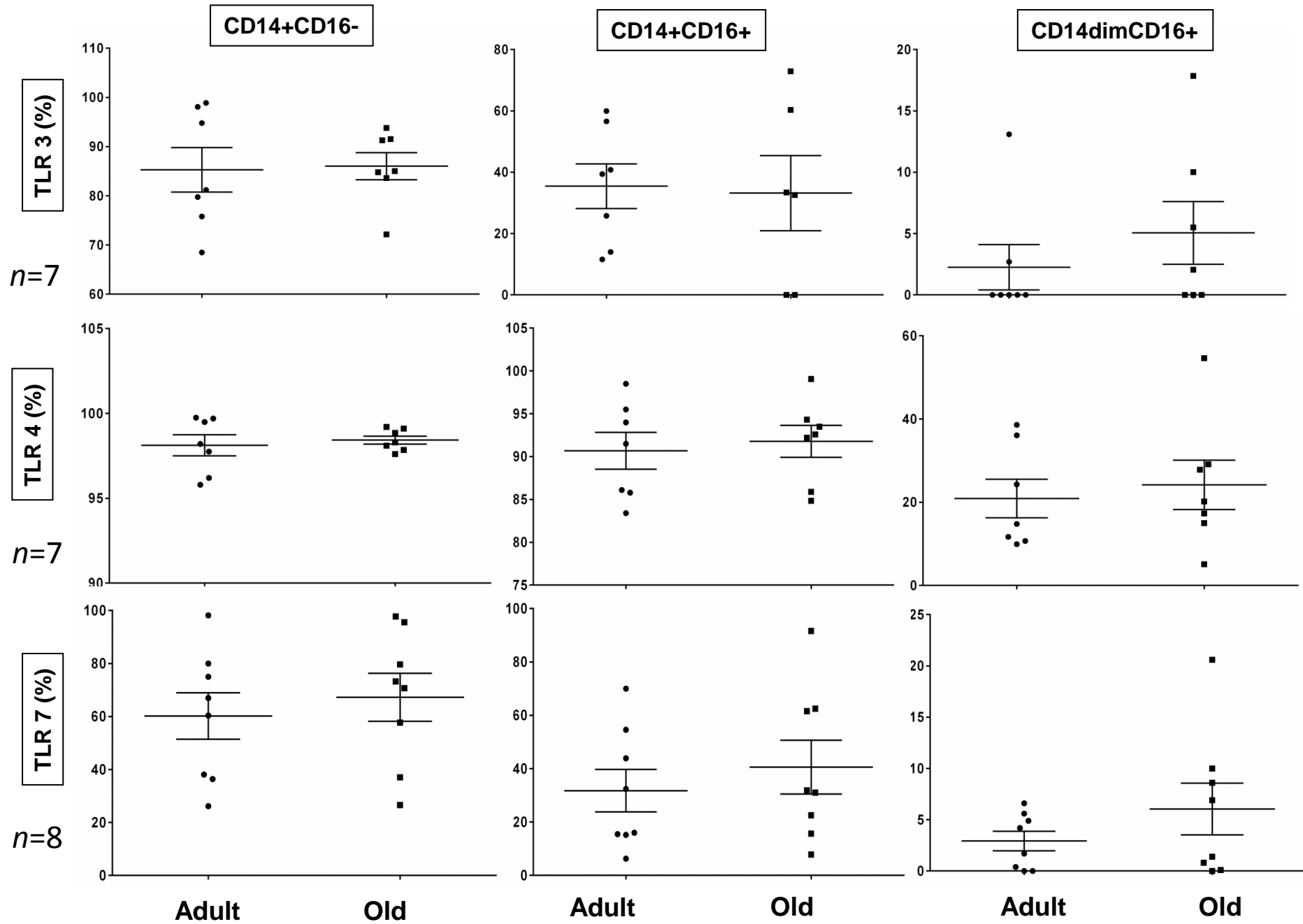

Supplement: Supplementary file 1 [file acel0014-0421-sd1.zip › SuppInform Figure 2 rev.pdf]

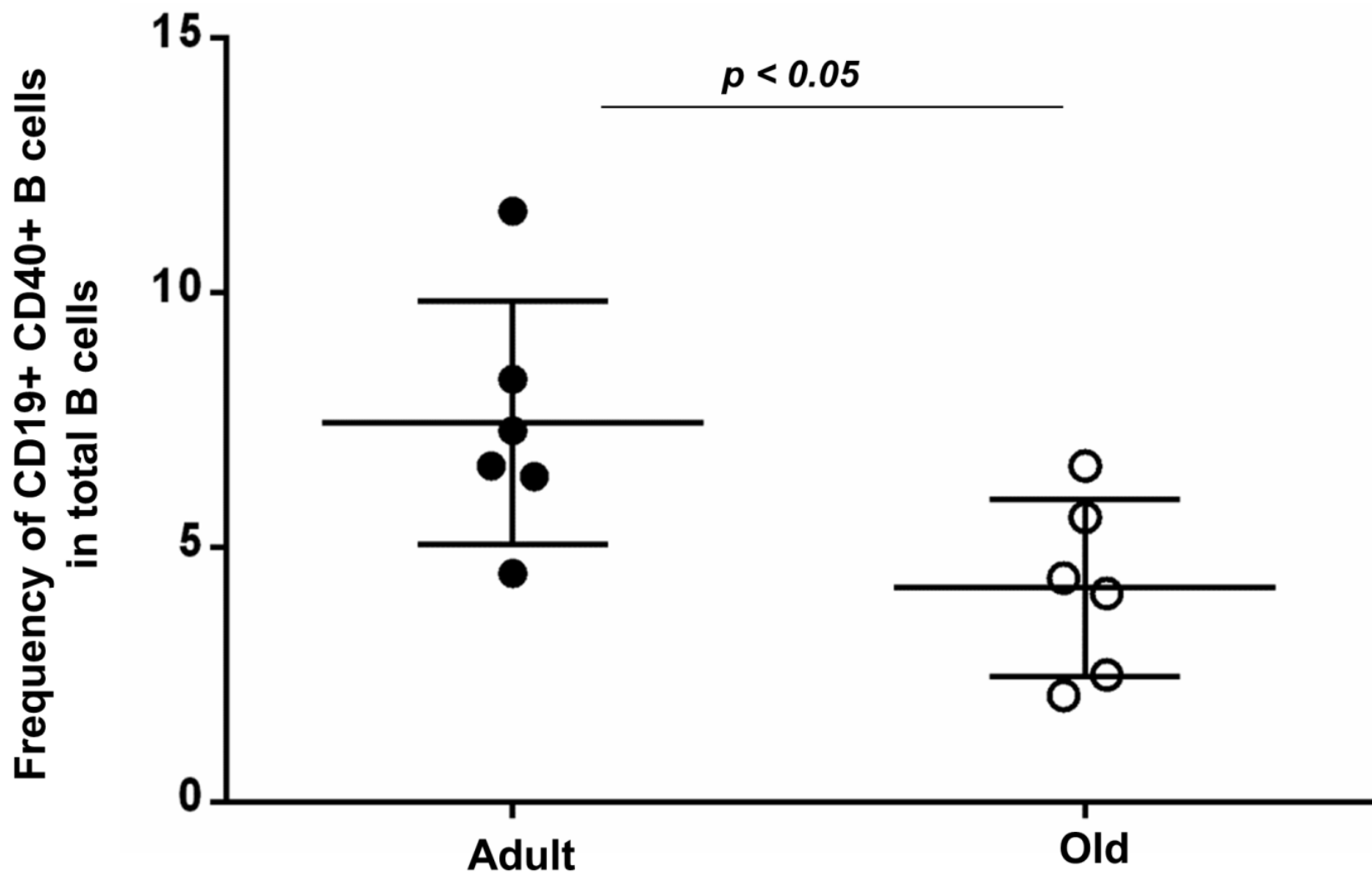

Supplement: Supplementary file 1 [file acel0014-0421-sd1.zip › SuppInform Figure 3 rev.pdf]
